# Opioidergic activation of the descending pain inhibitory system underlies placebo analgesia

**DOI:** 10.1126/sciadv.adp8494

**Published:** 2025-01-15

**Authors:** Hiroyuki Neyama, Yuping Wu, Yuka Nakaya, Shigeki Kato, Tomoko Shimizu, Tsuyoshi Tahara, Mika Shigeta, Michiko Inoue, Kazunari Miyamichi, Natsuki Matsushita, Tomoji Mashimo, Yoshiki Miyasaka, Yi Dai, Koichi Noguchi, Yasuyoshi Watanabe, Masayuki Kobayashi, Kazuto Kobayashi, Yilong Cui

**Affiliations:** ^1^ Laboratory for Biofunction Dynamics Imaging, RIKEN Center for Biosystems Dynamics Research, 6-7-3 Minatojima-Minamimachi, Chuo-ku, Kobe, Hyogo 650-0047, Japan.; ^2^ Multiomics Platform, Center for Cancer Immunotherapy and Immunobiology, Kyoto University, Yoshida-Konoe-cho, Sakyo-ku, Kyoto 606-8501, Japan.; ^3^ Department of Pharmacology, Nihon University School of Dentistry, 1-8-13 Kanda Surugadai, Chiyoda-ku, Tokyo 101-8310, Japan.; ^4^ Department of Molecular Genetics, Fukushima Medical University Institute of Biomedical Sciences, 1 Hikariga-oka, Fukushima 960-1295, Japan.; ^5^ Laboratory for Comparative Connections, RIKEN Center for Biosystems Dynamics Research, 2-2-3 Minatojima-Minamimachi, Chuo-ku, Kobe, Hyogo 650-0047, Japan.; ^6^ Division of Laboratory Animal Research, Aichi Medical University School of Medicine, 1-1 Yazakokarimata, Nagakute, Aichi 480-1195, Japan.; ^7^ Division of Animal Genetics, Laboratory Animal Research Center, Institute of Medical Science, The University of Tokyo, 7-3-1 Hongo, Bunkyo-ku, Tokyo 113-0033, Japan.; ^8^ Laboratory of Reproductive Engineering, Institute of Experimental Animal Sciences, Osaka University Medical School, 2-2 Yamadaoka, Suita, Osaka 565-0871, Japan.; ^9^ Department of Anatomy and Neuroscience, Hyogo Medical University, 1-1 Mukogawa, Nishinomiya, Hyogo 663-8501, Japan.; ^10^ Laboratory for Brain-Gut Homeostasis, Hyogo Medical University, 1-1 Mukogawa, Nishinomiya, Hyogo 663-8501, Japan.; ^11^ Laboratory for Pathophysiological and Health Science, RIKEN Center for Biosystems Dynamics Research, 6-7-3 Minatojima-Minamimachi, Chuo-ku, Kobe, Hyogo 650-0047, Japan.

## Abstract

Placebo analgesia is caused by inactive treatment, implicating endogenous brain function involvement. However, the neurobiological basis remains unclear. In this study, we found that μ-opioid signals in the medial prefrontal cortex (mPFC) activate the descending pain inhibitory system to initiate placebo analgesia in neuropathic pain rats. Chemogenetic manipulation demonstrated that specific activation of μ-opioid receptor–positive (MOR^+^) neurons in the mPFC or suppression of the mPFC–ventrolateral periaqueductal gray (vlPAG) circuit inhibited placebo analgesia in rats. MOR^+^ neurons in the mPFC are monosynaptically connected and directly inhibit layer V pyramidal neurons that project to the vlPAG via GABA_A_ receptors. Thus, intrinsic opioid signaling in the mPFC disinhibits excitatory outflow to the vlPAG by suppressing MOR^+^ neurons, leading to descending pain inhibitory system activation that initiates placebo analgesia. Our results shed light on the fundamental neurobiological mechanism of the placebo effect that maximizes therapeutic efficacy and reduces adverse drug effects in medical practice.

## Teaser

The placebo effect can relieve pain by activating the brain’s pain inhibition system via endogenous opioidergic signals.

## INTRODUCTION

Placebo effects are beneficial outcomes caused by ineffective sham treatment. Placebo effects exist in all clinical settings and affect therapeutic outcomes (*1*). However, their clinical applications have been limited because the underlying neurobiological mechanism remains unclear. Placebo analgesia is an actively studied placebo effect and a prime illustration of how the mind influences brain functions. The underlying mechanism of placebo analgesia involves activating the intrinsic pain inhibitory system by higher-order psychological processes (*2*). Neuroimaging studies in humans have demonstrated that hierarchical brain regions and neurochemical systems, such as dopaminergic and opioidergic systems, are involved in placebo analgesia (*3*, *4*). Endogenous opioidergic systems at multiple brain levels have been implicated in placebo analgesia, including the dorsolateral prefrontal cortex (dlPFC), rostral anterior cingulate cortex (rACC), amygdala, nucleus accumbens, and brainstem (*3*–*5*). The μ-opioid signal in the frontal regions, such as the rACC, has been proposed to drive the descending pain inhibitory or facilitatory system in the brainstem for placebo analgesia (*5*–*7*). However, the fundamental molecular and neuronal mechanisms underlying placebo analgesia remain unclear owing to the lack of comparable neuroimaging results from animal studies that allow mechanistic exploration using molecular, cellular, and genetic manipulations.

Placebo analgesia is caused by expectancy and conditioning in humans and rodents (*4*). Although the placebo effect in animal models is inconsistent across studies (*8*), we have successfully observed placebo analgesia following pharmacological conditioning paired with an analgesic in a rat model of neuropathic pain (*9*). Using small-animal neuroimaging analysis, we identified placebo analgesia–related brain activities that closely resemble those in human neuroimaging studies (*4*, *10*) and demonstrated that endogenous μ-opioid signal-dependent functional coupling between the medial prefrontal cortex (mPFC) and periaqueductal gray (PAG) was enhanced in response to conditioning-induced placebo analgesia (*9*). The μ-opioid receptor (MOR) is expressed in GABAergic interneurons in the mPFC and regulates the excitatory outflow of the PFC (*7*, *11*). However, the mPFC is connected to various brain regions and is engaged in diverse, complicated functions (*12*, *13*); thus, whether and how μ-opioid signaling in the mPFC drives the intrinsic descending pain modulatory system to facilitate or inhibit conditioning-induced placebo analgesia is unknown.

To address this issue, we developed genetically engineered rats expressing Cre recombinase under the control of the MOR promoter, in which the neuronal activity of MOR-expressing neurons can be specifically manipulated by optogenetic or chemogenetic methods. Chemogenetic activation of MOR-positive (MOR^+^) neurons in the mPFC abolished pharmacological conditioning-induced placebo analgesia in chronic neuropathic pain rats. Similarly, chemogenetic suppression of the neuronal circuit from the mPFC to the ventrolateral periaqueductal gray (vlPAG) also blocked the placebo analgesia. We also found that MOR^+^ neurons in the mPFC are monosynaptically connected with layer V pyramidal neurons that project to the vlPAG and predominantly inhibit those pyramidal neurons via GABA_A_ receptors. Here, we provide insight into the fundamental neurobiological basis of placebo analgesia and demonstrate that MOR^+^ neurons in the mPFC disinhibit excitatory outflow to the vlPAG, resulting in activation of the descending pain inhibitory system that initiates placebo analgesia.

## RESULTS

### Generation of MOR-Cre knock-in rat

**Fig. 1.**
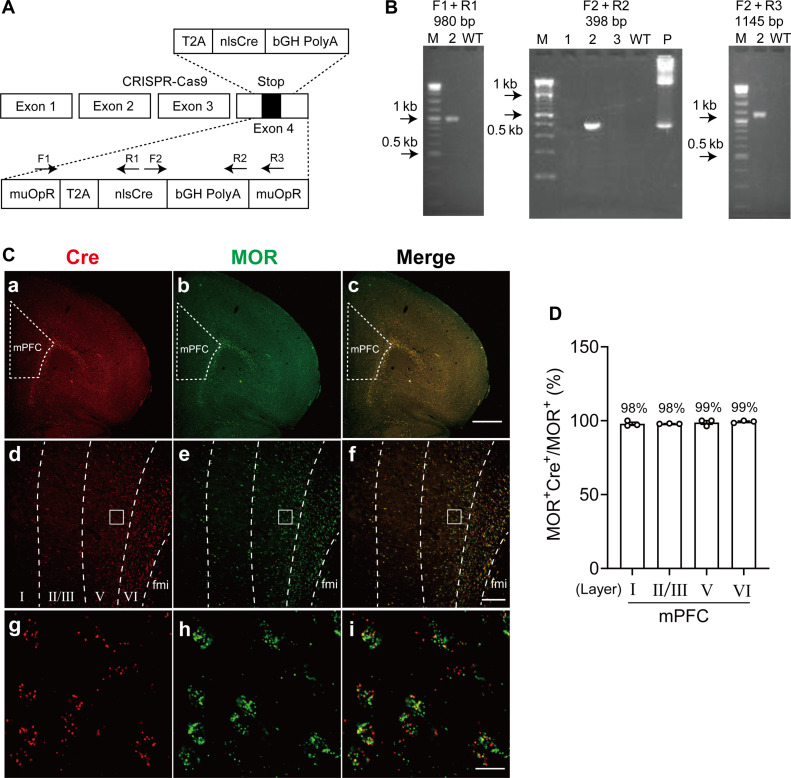
MOR^+^ neurons in the mPFC modulate placebo analgesia. (**A**) Targeting strategy for inserting *nlsCre*. (**B**) Gel images for genotyping of MOR-Cre KI rat. M, marker; P, positive control. (**C**) RNAscope fluorescent images of *MOR* and *Cre* coexpression in the mPFC. (a to c) Low magnification images for (a) *Cre*, (b) *MOR*, and (c) coexpression. (d to f) Images showing separative lines for cortical layers in mPFC. (g to i) High-magnification images for *Cre* (g), *MOR* (h), and coexpression (i). fmi, forceps minor of the corpus callosum. (**D**) Percentage of coexpression with MOR^+^ and *Cre*-positive cells in each cortical layer. Scale bars, 1000 μm (c), 200 μm (f), and 20 μm (i). WT, wild type.

To functionally manipulate MOR^+^ neuron activity in vivo, we designed and developed genetically engineered MOR-Cre rats, in which a cDNA encoding *Cre* recombinase was inserted into exon 4 of the *MOR* gene using CRISPR-Cas9 technology (Fig. 1A). The MOR-Cre Knock-in (KI) rats did not show any apparent malformations and were healthy and fertile. Polymerase chain reaction (PCR) using genomic DNA from tail clips of transgenic rats validated correct fragment size and sequence for internal 5′ and 3′ junction of the transgene insertion into the target locus (Fig. 1B). To confirm that *Cre* recombinase is specifically expressed in MOR^+^ neurons, we performed RNAscope in situ hybridization (ISH) with probes targeting *Cre* and *MOR* [Advanced Cell Diagnostics Inc. (ACDBio), Newark, CA]. As shown in fig. S1, *Cre* signals were observed in widespread brain regions consistent well with the MOR distribution as previously reported, such as the olfactory bulb, cerebral cortex, striatum, hippocampus, habenula, thalamus, PAG, and parabrachial nucleus (*14*, *15*). The clear *Cre* signals were scattered throughout the cerebral cortex, including the mPFC (Fig. 1C and fig. S1Ba). In the striatum, dense *Cre* signals were concentrated in unique patch patterns corresponding to the striosomes, which are well known to be enriched with MOR expression (fig. S1Bb) (*16*–*18*). Double RNAscope ISH with probes targeting *Cre* and *MOR* (gene symbol: *Oprm1*) showed that *Cre* signals were almost wholly colocalized with *MOR* in the habenula, interpeduncular nucleus, and striatum (fig. S2, A to C) (*14*, *15*, *19*). Similarly, most of MOR^+^ neurons in the mPFC coexpressed *Cre* and were consistent across cortical layers (Fig. 1C). As shown in Fig. 1D, the percentage of coexpression in each cortical layer of the mPFC was 97.9, 98.0, 98.8, and 99.3%, respectively.

### MOR^+^ neurons in the mPFC modulate neuropathic pain

**Fig. 2.**
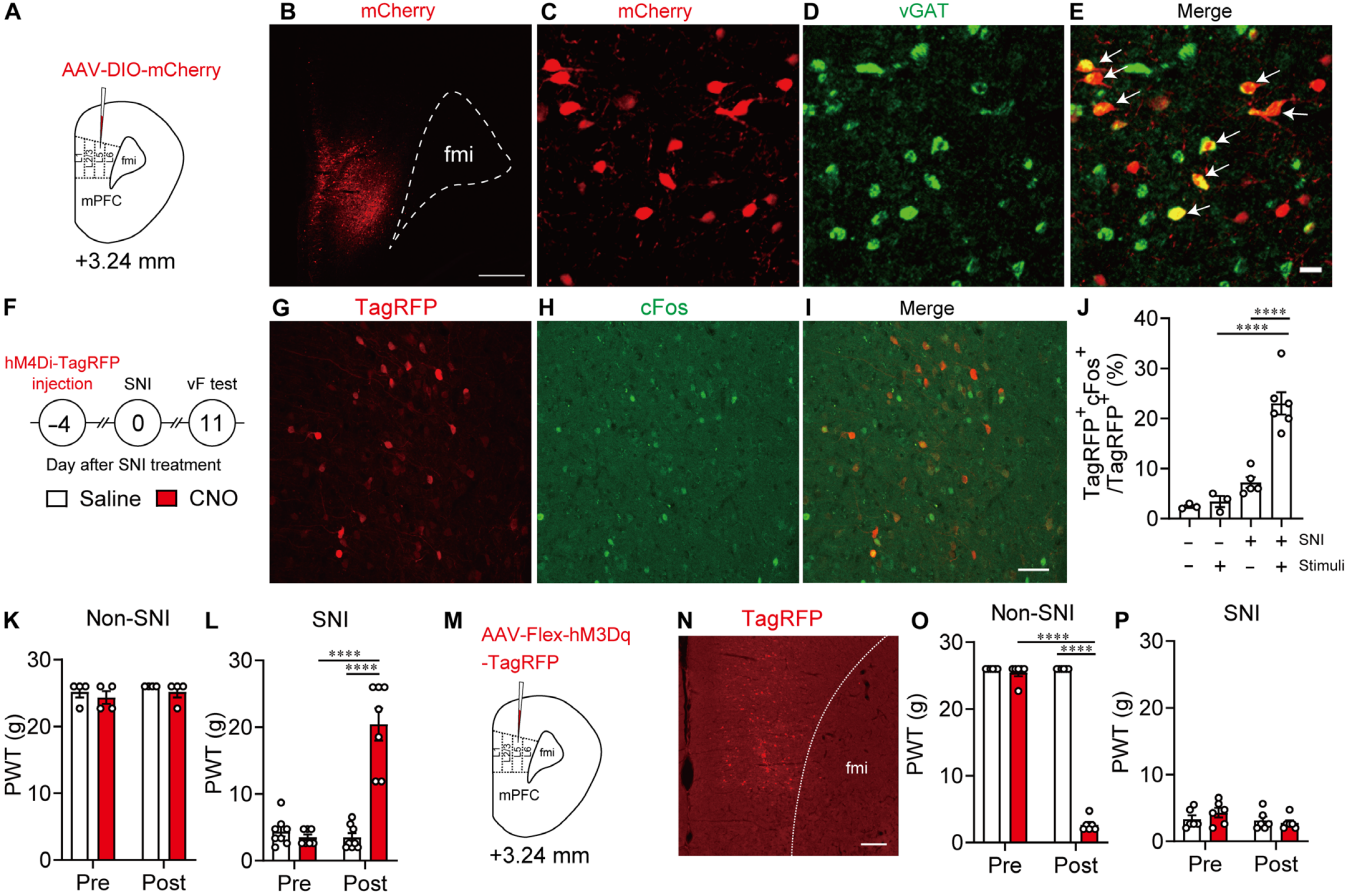
MOR^+^ neurons in the mPFC modulate nerve injury–induced pain. (**A**) Schematic diagram for AAV injection. (**B**) Representative coronal image showing MOR^+^ neurons in the mPFC labeled with mCherry (red). Scale bar, 500 μm. (**C** to **E**) Combined fluorescent immunostaining and fluorescent ISH images showing *vGAT* [green, (D)] expression in MOR^+^ neurons [mCherry, red, (C)] in the mPFC. White arrows in (E) indicate coexpression of *vGAT* in the MOR^+^ neurons. Scale bar, 20 μm. (**F**) Schematic diagram of chemogenetic manipulation of MOR^+^ neurons in the mPFC of MOR-Cre KI rat. (**G** to **I**) Double immunostaining images showing cFos [green, (H)] expressed in MOR^+^ neurons [TagRFP, red, (G)] in the mPFC. Scale bar, 50 μm. (**J**) Percentage of cFos^+^ neurons among MOR^+^ neurons. (**K**) Changes in paw withdrawal threshold (PWT) after saline and clozapine *N*-oxide (CNO) injection in non–spared nerve injury (SNI) rats microinjected with AAV-Flex hM4Di-TagRFP. (**L**) Changes in PWT after injection of saline and CNO in SNI rats microinjected with AAV-Flex-hM4Di-TagRFP. (**M**) Experimental procedure. (**N**) Immunostaining image of MOR^+^ neurons (TagRFP, red). Scale bar, 200 μm. (**O**) Changes in PWT after saline and CNO injection in non-SNI rats microinjected with AAV-Flex-hM3Dq-TagRFP. (**P**) Changes in PWT after saline and CNO injection in non-SNI rats microinjected with AAV-Flex-hM3Dq-TagRFP. ****P < 0.0001. All statistical information is presented in table S2.

To clarify whether and how MOR^+^ neurons in the mPFC modulate pain, we locally manipulated MOR^+^ neuron activity in the mPFC region of MOR-Cre KI rats with neuropathic pain using a chemogenetic method and evaluated pain behavior in response to nociceptive stimulation and related MOR^+^ neuron activity. We microinjected adeno-associated virus (AAV)–DIO–mCherry, AAV-Flex-hM4Di-TagRFP, or AAV-Flex-hM3Dq-TagRFP into layer V of the right prelimbic area of MOR-Cre KI rats, which is the major region of mPFC excitatory outflow (Fig. 2, A, B, M, and N) (*20*). Two weeks later, mCherry-positive (mCherry^+^) neurons were observed around the layer V mPFC and in layer II/III, indicating Cre-dependent recombination of the transgene in MOR-Cre KI rats (Fig. 2B). As shown in Fig. 2 (C to E), most mCherry^+^ neurons around layer V mPFC contained a vesicular GABA transporter (*vGAT*) (69.4 ± 2.4%, 58.3 ± 16.9 cells from four rats), indicating that MOR is largely expressed in the GABAergic interneurons, at least in the layer V mPFC, as previously reported (*21*). *Cre* and TagRFP^+^ neurons were not detected in wild-type rats (fig. S3).

To induce chronic neuropathic pain in MOR-Cre KI rats, we induced spared nerve injury (SNI) on the left hindlimb (Fig. 2F), which has been widely used as a neuropathic pain animal model with persistent and stable pain threshold for several weeks (*22*–*24*). In these SNI rats, cFos and TagRFP double-positive neurons were significantly increased in the right mPFC in response to pain stimuli to the left hindpaw 11 days after SNI treatment but not after sham treatment. These results indicate that MOR^+^ neurons in the mPFC were activated by pain stimuli (Fig. 2, G to J). Meanwhile, chemogenetic inhibition of MOR^+^ neurons in the mPFC significantly suppressed pain hypersensitivity [increased paw withdrawal threshold (PWT)] after intraperitoneal (ip) injection (1 mg/kg) of clozapine *N*-oxide (CNO) in SNI rats expressing AAV-Flex-hM4Di-TagRFP in MOR^+^ neurons in the mPFC but not in non-SNI rats (Fig 2, K and L). In contrast, chemogenetic activation of MOR^+^ neurons by CNO (1 mg/kg) enhanced pain responses in non-SNI rats expressing AAV-Flex-hM3Dq-TagRFP in MOR^+^ neurons in the mPFC but not in SNI rats (Fig. 2, O and P). Chemogenetic activation of AAV-Flex-hM3Dq-TagRFP–expressing MOR^+^ neurons in the mPFC was confirmed by cFos immunohistochemistry (fig. S4, A to C) and unit recording (fig. S4, D and E). Overall, MOR^+^ neurons in the mPFC are activated in response to pain stimuli, and specific inhibition of this activity can produce an analgesic effect.

### MOR^+^ neurons in the mPFC modulate placebo analgesia

**Fig. 3.**
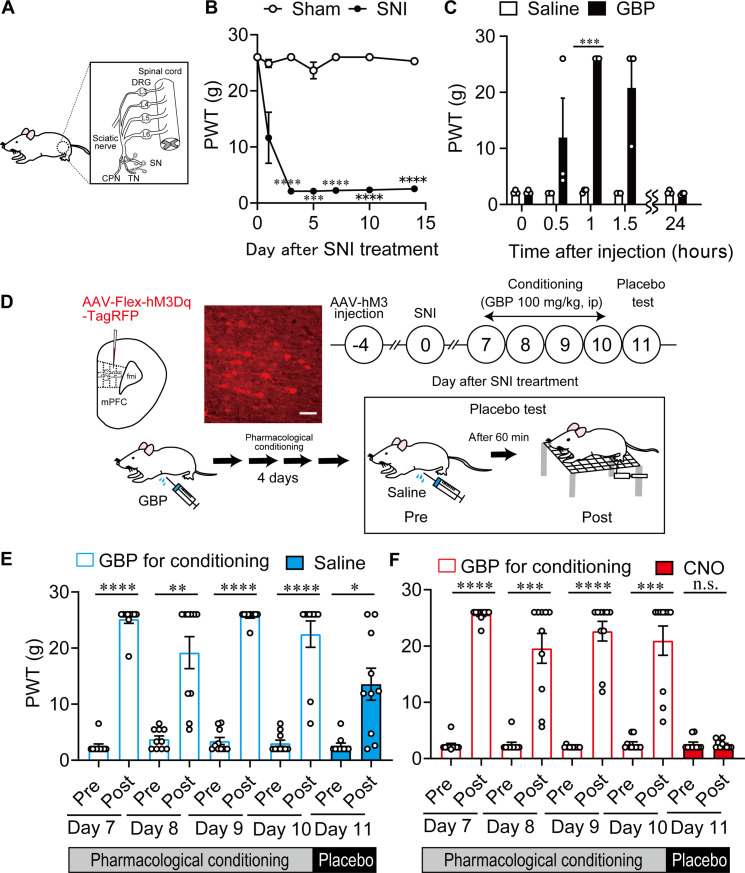
MOR^+^ neurons in mPFC modulate placebo analgesia. (**A**) Configuration of SNI. TN, tibial nerve; SN, sural nerve; CPN, common peroneal nerve; DRG, dorsal root ganglion. (**B**) Long-lasting pain hypersensitivity in SNI treatment. (**C**) Time-dependent changes in antihypersensitivity effect of GBP hydrochloride (100 mg/kg, intraperitoneal) injection. (**D**) Schematic diagram of designer receptors exclusively activated by designer drug (DREADD) experiment for placebo analgesia. Scale bar, 50 μm. (**E**) Changes in PWT after GBP injection for conditioning and saline injection for placebo test in SNI rats microinjected with AAV-Flex-hM3Dq-TagRFP. (**F**) Changes in PWT after GBP injection for conditioning and CNO injection for placebo test in SNI rats microinjected with AAV-Flex-hM3Dq-TagRFP. *P < 0.05, **P < 0.01, ***P < 0.001, and ****P < 0.0001. All statistical information is presented in table S2. n.s., not significant.

Subsequently, we examined whether and how MOR^+^ neurons in the mPFC modulate placebo analgesia. As previously reported, pain hypersensitivity was significantly increased (decreased PWT) after SNI treatment and remained stable for at least 2 weeks (Fig. 3, A and B). In these SNI rats, placebo analgesia was induced by pharmacological conditioning, in which gabapentin (GBP) administration (unconditioned stimulus) was paired with intraperitoneal injection (conditioned stimulus) for four consecutive days (Fig. 3D), as previously reported (*9*). For the conditioning procedure, the temporal profile of GBP-evoked analgesic effects was examined in the SNI rats first. As shown in Fig. 3C, pain responses tended to decrease at 30 min, and pain responses significantly reduced at 60 min after the intraperitoneal injection of GBP (100 mg/kg). Since such analgesic effects completely vanished 24 hours after the GBP injection, GBP was injected every day as an unconditioned stimulus and pain behavior were evaluated 1 hour after each injection. During the conditioning phase, pain responses were significantly reduced by each daily injection of GBP (unconditioned stimulus), indicating that the unconditioned stimulus results in effective analgesia to SNI rats. The pain threshold before each daily injection of GBP was also carefully evaluated, and we found that pain hypersensitivity in the SNI rats was preserved throughout the conditioning experiment. On the test day (day 5), pain responses were significantly reduced by intraperitoneal injection of saline as a placebo, indicating conditioning-induced placebo analgesia in these rats (Fig. 3E, blue bar, increased PWT). In contrast, chemogenetic activation of AAV-Flex-hM3Dq-TagRFP–expressing MOR^+^ neurons in the mPFC inhibited placebo analgesia after intraperitoneal injection of CNO instead of saline (Fig. 3F, red bar), but this was not observed in rats expressing AAV-Flex-TagRFP (control vector) (fig. S5). Overall, activating MOR^+^ neurons in the mPFC disturbs placebo analgesia.

### mPFC-vlPAG circuit modulates placebo analgesia

**Fig. 4.**
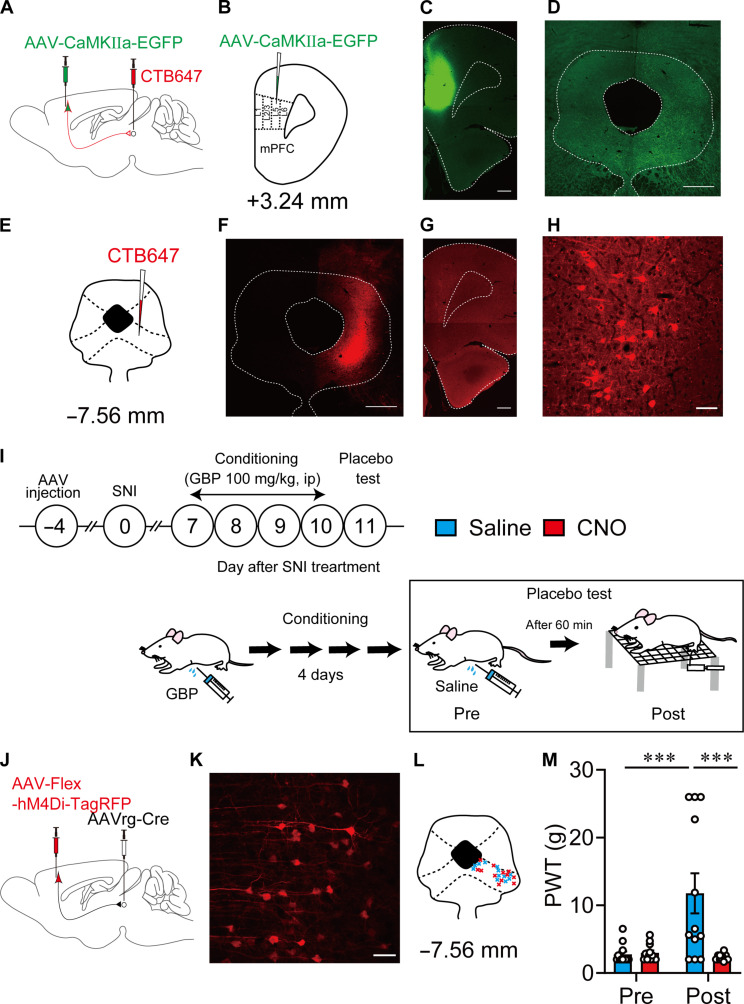
mPFC-vlPAG circuit modulates placebo analgesia. (**A** and **B**) Schematic diagram for anterograde (AAV-CaMKIIα-EGFP) and retrograde (CTB647) labeling of the mPFC-vlPAG circuit in wild-type rats. (**C** and **D**) Images show AAV injection side in the mPFC and the projection fiber in the vlPAG that was anterogradely labeled. Scale bars, 500 μm. (**E** to **H**) Retrograde somatic labeling of layer V pyramidal neurons in the mPFC, in which CTB647 was injected into the vlPAG. Scale bars, 500 μm (F), 500 μm (G), and 50 μm (H). (**I**) Experimental procedures for pharmacological conditioning-induced placebo analgesia and chemogenetic manipulation. (**J**) Schematic diagram for chemogenetic manipulation of mPFC-vlPAG circuit using AAV-Flex-hM4Di-TagRFP. (**K**) High-magnification image shows the expression of AAV-Flex-hM4Di-TagRFP in layer V pyramidal neurons that project into the vlPAG (blue, 4′,6-diamidino-2-phenylindole; red, TagRFP). Scale bar, 50 μm. (**L**) Retrograde AAV (AAVrg) injection points in the vlPAG. (**M**) Changes in PWT after saline and CNO injection for evaluating placebo analgesia. ***P < 0.001. All statistical information is presented in table S2.

The mPFC sends monosynaptic excitatory projections into vlPAG, a key node nucleus of the descending pain inhibitory system (*6*), to modulate pain processing (*25*). Thus, we verified the functional role of the mPFC-vlPAG circuit in placebo analgesia. First, we confirmed the anatomical projections from the mPFC to the vlPAG. An anterograde tracing experiment showed that abundant enhanced green fluorescent protein (EGFP)–expressing fibers were observed in the vlPAG 4 weeks after the microinjection of AAV-CaMKIIa-EGFP into the layer V mPFC to express EGFP in projection pyramidal neurons under the control of the glutamatergic-specific calmodulin kinase (CaMKIIa) promoter (Fig. 4, A to D). Furthermore, retrogradely traced cholera toxin subunit B 647 (CTB647) was predominantly observed in layer V pyramidal neurons of the mPFC after CTB647 microinjection into the vlPAG (Fig. 4, E to H). These results indicated that layer V pyramidal neurons in the mPFC send monosynaptic excitatory projections to the vlPAG (*25*). The layer V pyramidal neurons rarely overlapped with MOR^+^ neurons (1.2 ± 0.3%) (fig. S6).

Subsequently, we examined the functional role of the mPFC-vlPAG circuit in neuropathic pain modulation. To specifically manipulate the mPFC-vlPAG circuit activity, we concomitantly microinjected retrograde AAV (AAVrg)–Cre into the vlPAG and AAV-Flex-hM4Di-TagRFP or AAV-DIO-hM3Dq-mCherry into layer V of the right prelimbic area of the mPFC in wild-type rats (Fig. 4, I and J, and fig. S7, A and E). Thus, AAV-DIO-hM3Dq-mCherry or AAV-Flex-hM4Di-TagRFP was specifically expressed in layer V pyramidal neurons (Fig. 4, K and L, and fig. S7, B and F). Chemogenetic activation of the mPFC-vlPAG circuit by CNO injection in rats expressing AAV-DIO-hM3Dq-mCherry significantly suppressed pain hypersensitivity in SNI rats (fig. S7D). However, it did not produce an analgesic effect in saline-injected control rats (fig. S7C). In contrast, chemogenetic suppression of the mPFC-vlPAG circuit by CNO injection in rats expressing AAV-Flex-hM4Di-TagRFP significantly enhanced pain hypersensitivity in non-SNI rats but not in SNI rats (fig. S7, G and H). Consistent with previous reports (*25*–*27*), these results indicated that the mPFC-vlPAG circuit modulates physiological and pathophysiological pain processing by innervation of the downstream descending pain inhibitory system.

Last, we confirmed whether mPFC-vlPAG projection modulates conditioning-induced placebo analgesia. After four consecutive days of conditioning, chemogenetic inhibition of the mPFC-vlPAG circuit specifically expressing AAV-Flex-hM4Di-TagRFP significantly reduced the analgesic effect (deceased PWT) in rats injected with CNO instead of saline (Fig. 4M). The analgesic effect did not change in analogous experiments using AAV-Flex-TagRFP as the control virus (fig. S8). These results demonstrated that inhibiting the mPFC-vlPAG circuit disturbs placebo analgesia in SNI rats. Overall, suppressing MOR^+^ neuronal activity and activating the mPFC-vlPAG circuit may underlie placebo analgesia in SNI rats. Thus, we further investigated whether and how MOR^+^ neurons regulate the mPFC-vlPAG circuit.

### Monosynaptic connections from MOR^+^ neuron to vlPAG-projecting layer V pyramidal neuron in the mPFC

**Fig. 5.**
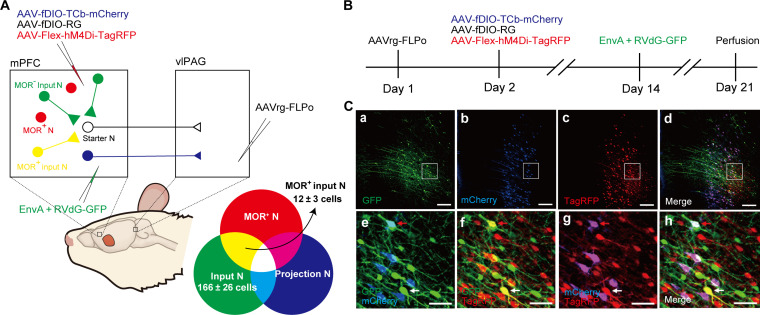
Monosynaptic connection between MOR^+^ neurons and mPFC-vlPAG circuit. (**A**) Schematic diagram for rabies virus–based retrograde trans-synaptic tracing. (**B**) Timeline of virus injection for rabies virus–based retrograde trans-synaptic tracing. (**C**) Representative fluorescent images show rabies virus–based retrograde trans-synaptic tracing. (Ca to Ch) mPFC coronal sections show starter neurons labeled in white [expressing GFP (green), mCherry (blue), and TagRFP (red)]; input neurons in green (expressing GFP); projection neurons in magenta, merged by mCherry (blue) and TagRFP (red); and MOR^+^ neurons in red (TagRFP). (Ce to Ch) Higher-magnification images of (Ca) to (Cd). Note that neurons labeled in yellow [merged by GFP (green) and TagRFP (red)] in the images (Cd) and (Cf) indicate MOR^+^ neurons monosynaptically connected with neurons that project into the vlPAG. Scale bars, 200 μm (Ca to Cd) and 50 μm (Ce to Ch).

First, we examined whether there are direct monosynaptic connections between MOR^+^ neurons and layer V pyramidal neurons in the mPFC that project into the vlPAG using a rabies virus–mediated retrograde trans-synaptic tracing method in MOR-Cre KI rats (*28*). We microinjected AAVrg-FLPo into the vlPAG, followed by the microinjection of a mixture of AAV-fDIO-TCb-mCherry and AAV-fDIO-RG into the mPFC layer V in MOR-Cre KI rats. To identify MOR^+^ neurons, AAV-Flex-hM4Di-TagRFP was microinjected into the mPFC layer V. Two weeks later, EnvA-pseudotyped glycoprotein-deleted rabies viruses expressing GFP (EnvA + RVdG-GFP) were microinjected into the mPFC layer V to initiate trans-synaptic tracing (Fig. 5, A and B). The layer 5 pyramidal neurons (Fig. 5Cb) that project into the vlPAG were identified by an antibody that can discriminate mCherry from TagRFP (Fig. 5Cc). Various GFP^+^ input neurons (Fig. 5Ca) were distributed in layer V and layer II/III of the mPFC, trans-synaptically labeled from starter neurons (Fig. 5, Cd and Ch; yellow neurons) among layer V pyramidal neurons (Fig. 5Cb). GFP^+^ input neurons coexpressed TagRFP but not mCherry (7.7 ± 2.7, four rats; Fig. 5, Cf and Ch), indicating that MOR^+^ neurons monosynaptically connected with layer V pyramidal neurons (Fig. 5, Ce and Ch; blue and white neurons) that project into the vlPAG. No mCherry or GFP expression was observed in rats without microinjection of AAVrg-FLPo into the vlPAG (fig. S9).

### MOR^+^ neurons predominantly inhibit excitatory outflow of mPFC to the vlPAG

**Fig. 6.**
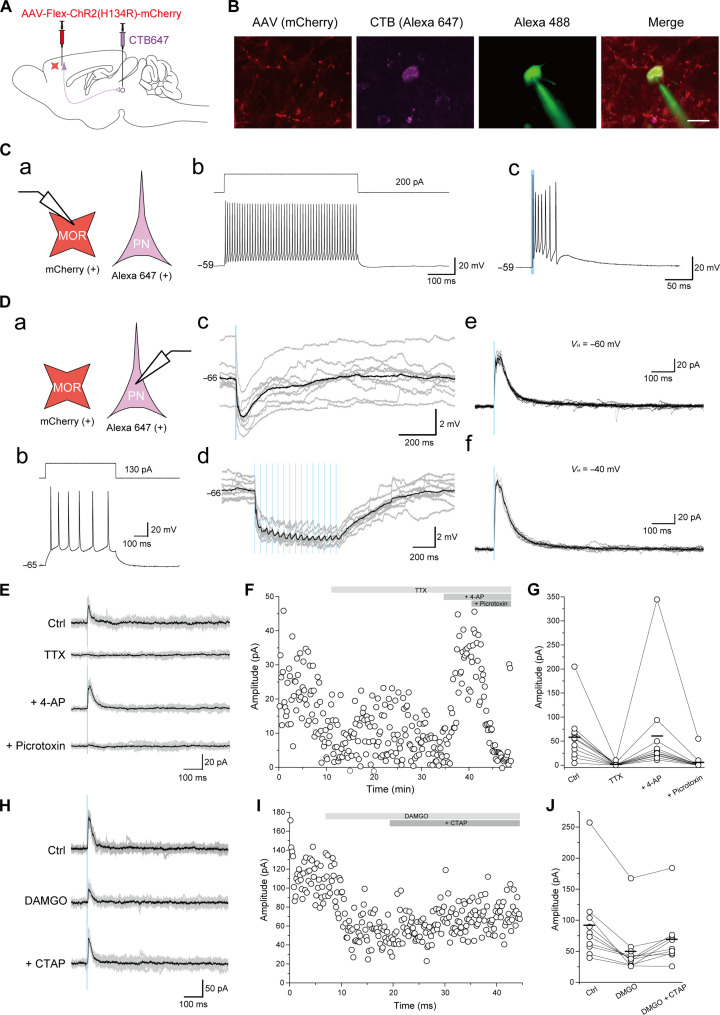
MOR^+^ neurons predominantly inhibit the excitatory outflow of the mPFC to the vlPAG. (**A**) Schematic diagram for optogenetic manipulation of MOR^+^ neurons and labeling layer V pyramidal neuron in the mPFC that projects into the vlPAG. (**B**) Images show representative MOR^+^ neurons (red), layer V pyramidal neuron (magenta, CTB647), and representative recording neurons (green, Alexa 488 contained in internal electrode solution). Scale bar, 20 μm. (**C**) Whole-cell patch-clamp recording from MOR^+^ neurons. (a) Schematic diagram. PN, pyramidal neurons. (b) Representative firing responses induced by current injection. (c) Representative firing responses induced by photostimulation. (**D**) Whole-cell patch-clamp recording from CTB647^+^ layer V pyramidal neuron that projects into the vlPAG. (a) Schematic diagram. (b) Representative firing responses induced by current injection. (c to d) Current-clamp recording shows photostimulation-induced IPSP. (e to f) Voltage-clamp recording shows photostimulation-induced IPSC. (**E** to **G**) Voltage-clamp recording of IPSC from CTB647^+^ layer V pyramidal neuron without (Ctrl) and with tetrodotoxin (TTX); TTX and 4-AP; or TTX, 4-AP, and picrotoxin. (**H** to **J**) Voltage-clamp recording of IPSC from CTB647^+^ pyramidal neuron without (Ctrl) and with DAMGO or DAMGO and CTAP.

We examined how MOR^+^ neurons regulate layer V pyramidal neurons that project to the vlPAG using whole-cell patch-clamp recording in PFC slices from SNI rats 10 days after surgery. We concomitantly microinjected CTB647 into the vlPAG for retrograde labeling of layer V pyramidal neurons in the mPFC and AAV-Flex-hChR2(H134R)-mCherry into the mPFC layer V to express excitatory opsin, ChR2(H134R), and mCherry in MOR^+^ neurons in MOR-Cre KI rats (Fig. 6, A and B). Most mCherry^+^ neurons (87.0%, 20 of 23) showed high-frequency action potentials without apparent frequency accommodation (Fig. 6, Ca and Cc), suggesting that they were fast-spiking interneurons. In the mCherry^+^ fast-spiking interneurons, single blue laser irradiation induced multiple action potentials (81.0%, 17 of 21; Fig. 6Cc) or excitatory postsynaptic potentials (19.0%, 4 of 21). CTB647^+^ neurons (28 neurons from 10 rats) showed a representative layer V pyramidal neuron firing pattern (Fig. 6, Da and Db). Single blue laser irradiation induced inhibitory postsynaptic potentials (IPSPs) (0.5 ± 0.1 mV, *n* = 28). We further discovered that 15-train photostimulation-induced IPSP summation followed by a sustained hyperpolarization: 1.8 ± 0.3 mV in amplitude and 528.7 ± 84.3 ms from the onset to peak (*n* = 28; Fig. 6, Dc and Dd). Under the voltage-clamp condition, the inhibitory postsynaptic current (IPSC) amplitudes were 8.3 ± 3.0 pA (*n* = 28) at the holding potential of −60 mV and 56.0 ± 12.4 pA at −40 mV (*n* = 27; Fig. 6, De and Df). We then examined whether CTB647^+^ layer V pyramidal neurons received monosynaptic inhibitory inputs from mCherry^+^ neurons (Fig. 6, E to G). Applying 1 μM tetrodotoxin abolished blue light–evoked IPSCs from 63.6 ± 17.8 to 1.7 ± 1.1 pA (*n* = 11; Fig. 6, E to G). 4-Aminopyridine (4-AP; 1 mM) application recovered IPSCs to 64.7 ± 33.7 pA (*n* = 11; Fig. 6G), suggesting that photostimulation-evoked outward currents were mediated via monosynaptic connections. Outward currents were abolished by picrotoxin (100 μM; *n* = 11; Fig. 6G), indicating the involvement of GABA_A_ receptors. Bath application of 1 μM [D-Ala2, N-Me-Phe4, Gly5-ol]-Enkephalin (DAMGO), a MOR agonist, reduced the photostimulation-evoked IPSC amplitude from 92.0 ± 20.9 to 49.8 ± 14.1 pA, whereas 2 μM D-Phe-Cys-Tyr-D-Trp-Arg-Thr-Pen-Thr-NH2 (CTAP), a selective MOR antagonist, recovered the DAMGO-induced suppression of photostimulation-evoked IPSCs to 69.2 ± 14.4 pA (*n* = 10; Fig. 6, H to J). These results suggested that MOR^+^ neurons directly inhibit layer V pyramidal neurons, which project to the vlPAG via GABA_A_ receptors.

### mPFC-vlPAG circuit is essential for MOR^+^ neuron–mediated pain modulation

**Fig. 7.**
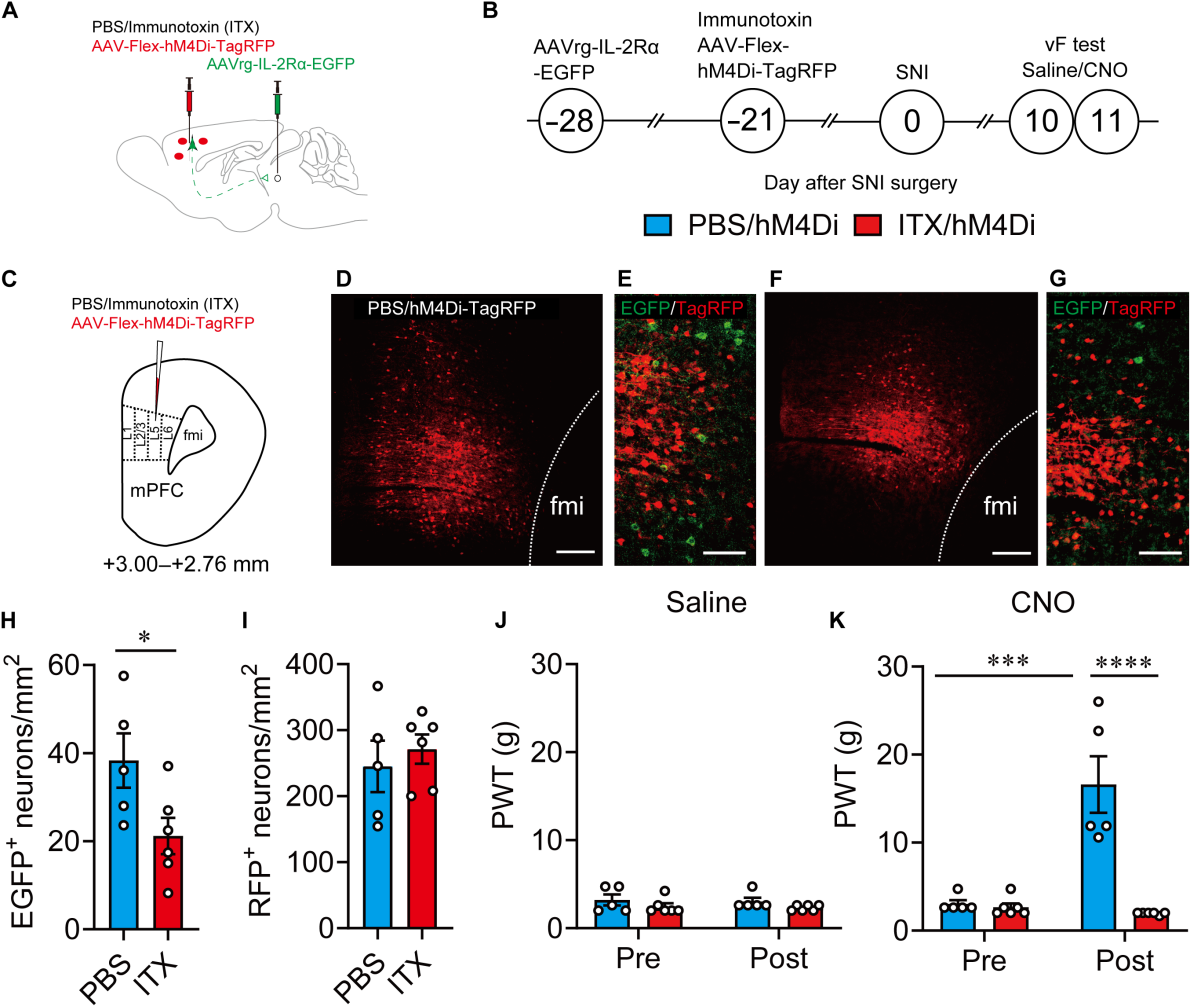
mPFC-vlPAG circuit is essential for MOR^+^ neuron–mediated pain modulation. (**A** to **C**) Schematic diagram for specific ablation of mPFC-vlPAG circuit using an ITX-mediated elimination method and the timeline for chemogenetic manipulation and behavior test. (**D** and **F**) Images show TagRFP expression in the mPFC in PBS- and ITX-treated rats. Scale bars, 200 μm. (**E** and **G**) High-magnification images showing TagRFP^+^ and GFP^+^ neurons. Scale bars, 100 μm. (**H**) The number of GFP^+^ neurons in PBS- and ITX-treated groups. (**I**) The number of TagRFP^+^ neurons in PBS- and ITX-treated groups. (**J** and **K**) Changes in PWT after saline (J) or CNO (K) injection in MOR Cre KI rat with PBS/hM4Di (light blue column) or ITX/hM4Di (red column). *P < 0.05, ***P < 0.001, and ****P < 0.0001. All statistical information is presented in table S2.

Last, to confirm whether the mPFC-vlPAG circuit is essential for MOR^+^ neuron–mediated pain modulation, we selectively ablated the layer V pyramidal neurons in the mPFC that projected into the vlPAG and examined pain behavior in response to specific manipulation of MOR^+^ neuron activity. Selective ablation of mPFC-vlPAG was conducted using an immunotoxin [ITX; anti-Tac(Fv)-PE38, gift from I. Pastan]–mediated neural circuit elimination approach in which the AAVrg encoding human interleukin-2 receptor α-subunit (IL-2Rα) and EGFP (AAVrg-IL-2Rα-EGFP) was microinjected into the vlPAG to retrogradely express IL-2Rα in the mPFC layer V pyramidal neurons, followed by the microinjection of recombinant ITX into the mPFC for selective ablation of target neurons (Fig. 7, A to D and F). Similar to our previous report (*29*), the number of EGFP-expressing layer V pyramidal neurons that project into the vlPAG was significantly reduced by the injection of ITX (5 ng/μl) compared with that after the injection of phosphate-buffered saline (PBS; Fig. 7, E, G, and H). However, the number of TagRFP-expressing MOR^+^ neurons in the surrounding area did not change between ITX- and PBS-injected rats (Fig. 7, E, G, and I), indicating that the mPFC-vlPAG circuit was selectively ablated. The chemogenetic suppression of MOR^+^ neurons in these rats revealed decreased pain hypersensitivity in PBS-injected rats expressing AAV-Flex-hM4Di-TagRFP in MOR^+^ neurons in the mPFC (Fig. 7, J and K). In contrast, this analgesic effect completely disappeared in ITX-injected rats (Fig. 7, J and K), indicating that the mPFC-vlPAG circuit is essential for MOR^+^ neuron–mediated pain modulation.

## DISCUSSION

We demonstrated the fundamental neurobiological mechanisms underlying placebo analgesia. Intrinsic opioid signaling in the mPFC disinhibits excitatory outflow to the vlPAG via the suppression of MOR^+^ neurons, leading to the activation of the descending pain inhibitory system. We provide evidence that (i) chemogenetic activation of MOR^+^ neurons in the mPFC inhibits pharmacological conditioning-induced placebo analgesia in rats, (ii) chemogenetic suppression of the mPFC-vlPAG circuit also blocks conditioning-induced placebo analgesia, and (iii) MOR^+^ neurons in the mPFC are monosynaptically connected and directly inhibit layer V pyramidal neurons that project to the vlPAG via GABA_A_ receptors. Placebo analgesia is evoked by an inert treatment that is ineffective, suggesting that an endogenous pain inhibitory system must be activated by higher-order neuropsychological processes such as expectations (*10*, *30*). Neuroimaging studies in humans have demonstrated that several prefrontal cortical regions, such as the rACC and dlPFC, are activated by placebo administration and subsequently recruit the downstream descending pain modulatory system involving the PAG/brainstem and rostroventromedial medullar to suppress pain (*3*–*5*, *31*, *32*). The functional coupling between these prefrontal regions and the PAG/brainstem is enhanced during placebo analgesia (*5*, *31*, *33*–*35*). The dlPFC in humans is one of the highest-order cortical regions involved in cognitive and affective components of pain modulation (*4*, *36*–*38*). The regional brain activity in the dlPFC is correlated with placebo-induced pain relief (*32*, *39*), and suppression of the dlPFC activity by transcranial magnetic stimulation abolished the placebo responses (*40*), suggesting that the activation of the dlPFC is essential for placebo analgesia. The dlPFC is activated during the anticipation phase of placebo analgesia (*5*), and a significant covariation of brain activity between the dlPFC and vlPAG predicts placebo responses (*41*), indicating that the dlPFC seems to be involved in the initiation of placebo response and drive descending pain modulatory system. Although controversies have been noted, based on the neural connection with the mediodorsal nucleus of the thalamus (*42*) and the functional similarity (*12*, *43*), the mPFC in rodents is considered to be homologous to the primate dlPFC (*44*–*46*). In rodents, the mPFC drives the descending pain inhibitory system via the vlPAG (*6*, *25*–*27*). Using small-animal neuroimaging analysis, we previously demonstrated that the activation of the mPFC is essential and that the functional coupling between the mPFC and vlPAG is increased in pharmacological conditioning-induced placebo analgesia in rats (*9*). These observations suggest that the current data can provide a fundamental mechanism of placebo analgesia and that the human placebo effect involves similar neurobiological processes.

Endogenous opioids interacting with MOR in hierarchical brain regions have been implicated in placebo analgesia, including the higher-order frontal cortex (*2*, *3*, *5*, *7*, *47*, *48*). In humans, pharmacological functional magnetic resonance imaging and positron emission tomography imaging studies have demonstrated that the brain activities in multilevel brain regions such as the dlPFC, rACC, PAG, and rostroventromedial medulla are changed by both opioid or placebo analgesia and are reversed by naloxone, a μ-opioid antagonist (*3*, *5*). The μ-opioid signaling activity in the rACC, ventromedial prefrontal cortex, nucleus accumbens, and PAG/brainstem is related to placebo analgesia (*3*, *47*, *49*, *50*) and that in the dlPFC is positively correlated with expectation for pain relief (*48*, *49*). The frontal area, such as the rACC, is activated by endogenous μ-opioid signal during placebo and subsequently drives descending pain inhibitory system in brainstem regions for the placebo analgesia (*5*, *7*, *47*, *48*, *50*). However, ACC activation was also observed with painful stimulation and suppression of such activity alleviated pain and related aversive responses, suggesting that increased excitatory outflow from frontal regions, such as the ACC, may also facilitate the descending pain facilitatory system in the brainstem (*51*, *52*). The neurons in the ACC comprise a complicated local network, including MOR^+^ neurons, and connect with varied brain regions to engage in multifunctional modulations, suggesting that neuronal-level investigation is necessary to investigate the fundamental mechanisms of where and how the endogenous μ-opioid signal activates the descending pain modulation system to inhibit or facilitate pain. MOR, a major opioid receptor related to opioidergic analgesia, is broadly expressed from the peripheral nerves to higher-order brain regions and has been extensively discussed as a key player in pain modulation (*19*, *47*, *48*, *53*, *54*). Recently, single-cell RNA sequencing analysis in the cortical regions has shown that MOR is expressed in both excitatory and inhibitory neurons, although the laminar characterization of these neurons is still inconsistent due to the limited spatial resolution (*55*, *56*). Using transgenic MOR-mCherry reporter mice, the identity and laminar distribution of MOR^+^ neurons have revealed that MOR^+^ excitatory neurons predominantly observed in the layer VIb, which integrates long-range intracortical information (*57*), appear across cortical layers (*21*, *58*, *59*). Meanwhile, MOR^+^ inhibitory neurons are distributed similarly across cortical layers and relatively decreased in the deep layer such as layers VIa and VIb (*21*, *55*, *58*). In the frontal area, MOR^+^ excitatory neurons account for approximately 30% of MOR^+^ neurons, which is lower than isocortical regions, such as the primary somatosensory cortex (*21*). Consistently, we also confirmed that approximately 70% of MOR^+^ neurons around layer V of the mPFC were GABAergic interneurons (Fig. 2, C to E). Given that exogenous and endogenous opioids in the frontal area show clear analgesia, the functional role of MOR^+^ excitatory and inhibitory neurons in the area remains unclear. Recently, optogenetic suppression of GABAergic interneurons in the mPFC has also been reported to facilitate excitatory outflow to the vlPAG, producing an analgesic effect in an animal model of neuropathic pain (*25*). Because MOR is known to couple with Gi/Go proteins that inhibit neuronal activity (*7*), opioid-induced analgesia in the mPFC is more likely attributed to the inhibition of MOR^+^ GABAergic interneurons. In the present study, we demonstrated that MOR^+^ neurons in the mPFC monosynaptically connect with layer V pyramidal neurons that project into the vlPAG and MOR scarcely expressed in that layer V projection neurons (fig. S6). Our data also clearly demonstrated that selective optogenetic activation of MOR^+^ neurons predominantly inhibits these neurons via GABA_A_ receptors (Fig. 6, E to G). Furthermore, chemogenetic activation or suppression of MOR^+^ neurons facilitates or inhibits pain behavior in neuropathic pain rats, respectively (Fig. 2, L and P). Moreover, we recently demonstrated that the microinfusion of a MOR antagonist into the mPFC suppresses placebo analgesia in rats (*9*). These observations suggest that endogenous μ-opioid signaling in the mPFC may suppress MOR^+^ GABAergic interneurons, leading to the disinhibition of excitatory outflow to the vlPAG mediated by the monosynaptic GABA_A_ receptor, which initiates placebo analgesia.

Placebo is induced by not only expectation but also classical conditioning (*60*), although the interaction between them remains incompletely understood. Since discovered by Russian physiologist Ivan Pavlov, classic Pavlovian conditioning has been extensively used in neurophysiological and psychological fields to explore fundamental brain functions, including the placebo response in experimental animals and humans. In the 1950s, classical conditioning was reported to induce a placebo response, and some expectancies were considered to be generated by classical conditioning, which was proposed as a mechanism of the placebo effects (*61*, *62*). Recently, a study clearly demonstrated that placebo response could be caused by classical conditioning independent of verbal suggestion–based expectations (*63*), suggesting that classical conditioning involves either conscious expectations or unconscious processes (without expectations) for driving the placebo effect (*64*). In experimental animals, Herrnstein (*65*) demonstrated for the first time that classic Pavlovian conditioning can induce a placebo effect in rats. Pursuing this line of concept, classical conditioning paired with some active drugs has been demonstrated to evoke placebo analgesia in acute pain model animals, such as morphine, fentanyl, and aspirin (*66*). However, the placebo analgesia in chronic pain animal models remains inconsistent across studies. In a chronic neuropathic pain model, McNabb *et al*. (*8*) reported that classical conditioning with an active analgesic failed to induce significant placebo analgesia. However, the main reason for the failed placebo analgesia is considered to be the complicated contextual cues they used as conditioned stimuli. In the study, McNabb *et al.* used quite complicated sensory cues as the conditioned stimuli, combining environmental, temporal, olfactory, visual, and tactile cues. Because learning deficits and impaired cognitive functions have been reported in chronic pain animals (*67*), the success rate of conditioning should be decreased in chronic pain animals when complicated and mixed sensory cues are used as conditioned stimuli. Accordingly, using a simple intraperitoneal injection as the conditioned stimulus, we successfully induced placebo analgesia in chronic neuropathic pain rats (*9*). Consistent with the result, placebo analgesia was also reported in neuropathic pain models by morphine pharmacological conditioning (*68*). Given that conditioning-based behavioral tests, such as the conditioned place preference test, have been widely used in chronic pain animals (*69*, *70*), these observations suggest that classical conditioning is sufficient to induce placebo analgesia even in chronic pain animals.

Distinct neuroanatomical and neurochemical systems seem to be involved in expectation-dependent and expectation-independent placebo analgesia, respectively (*71*). Expectation-dependent placebo analgesia is mediated by endogenous opioid signaling (*48*, *50*, *72*) and involves activation in the cognitive and executive frontal regions, such as the rACC and dlPFC (*73*), which drives descending pain modulatory system in the vlPAG and rostral ventromedial medulla (RVM) (*5*). Expectation for pain relief was positively correlated with μ-opioid activity in the dlPFC (*48*, *72*). In contrast, expectation-independent placebo analgesia is mediated by endogenous cannabinoids (*74*, *75*) and relies on neural activation in the pathway from the amygdala to the dorsolateral periaqueductal gray and RVM (*71*). In the present study, we found that μ-opioid signals in the mPFC activate descending excitatory projections into the vlPAG to evoke placebo analgesia following pharmacological conditioning with an active analgesic in chronic neuropathic pain rats. Pain relief in neuropathic pain animals activates the mesolimbic dopamine system (*76*), a critical reward system in the brain, indicating that expectation-like sensation may be produced by active analgesic-induced pain relief during conditioning phase in the present study, which may explain why the expectation-dependent neurobiological mechanisms were observed in the present study. Together, these observations suggest that placebo analgesia induced by pharmacological conditioning paired with active analgesic in neuropathic pain animals seems to be dependent on expectation. Recently, Chen *et al.* (*77*) reported that the expectation of placebo analgesia is mediated by a neural circuit from the ACC to the pontine nucleus, using a nonpharmacological conditioning-induced placebo animal model. Under physiological conditions, they demonstrated that the activity of ACC neurons projecting to the pontine nucleus is responsible for the expectation of pain relief from acute thermal nociception. Unfortunately, the involvement of the vlPAG in the expectation and the activation of the descending pain inhibitory system were not investigated in this study. Since neural plasticity in widespread brain regions has frequently been observed in chronic pain, distinguished neurobiological mechanisms of placebo analgesia between the physiological and chronic pain conditions is an open subject to address in the future, including the expectation.

Placebo effects exist in any medical treatment and affect therapeutic outcomes. The present study demonstrated the fundamental mechanisms of placebo analgesia in chronic pain condition that enable us to explore placebo effects in medical practice to maximize therapeutic efficacy and reduce adverse drug effects and tolerance. Opioidergic drugs are the first choice for alleviating chronic pain; however, several serious side effects, such as addiction, respiratory depression, and sedation, limit their clinical application. According to The Global Burden of Diseases, Injuries, and Risk Factors Study estimate, around 109,500 people died from opioid overdose in 2017 (*78*). An effective combination with the placebo effect may provide a practical solution to minimize opioid intake and reduce its side effects.

In conclusion, we demonstrated the fundamental neurobiological basis of placebo analgesia, i.e., MOR^+^ neurons in the mPFC activate the descending pain inhibitory system within the vlPAG via GABA_A_ receptors to initiate placebo analgesia. Unlike many other biomedical studies, most placebo research has been conducted in humans using noninvasive neuroimaging methods such as functional magnetic resonance imaging and positron emission tomography. On the basis of current neuroimaging studies in human, hierarchical brain regions and several neurochemical systems have been proposed as the underlying mechanisms of placebo analgesia. However, the lack of animal studies, especially in chronic pain animals, makes it difficult to understand the detailed neurobiological basis of the placebo effect and hinders its clinical applications. Recently, we have successfully established placebo analgesia in chronic neuropathic pain animals and identified placebo analgesia–related brain activities using small-animal neuroimaging analysis that is similar to that used in human placebo studies (*9*). In the present study, we further developed genetically engineered MOR-Cre rats and demonstrated the fundamental neurobiological mechanisms of placebo analgesia, especially in chronic pain, using neurophysiological, optogenetic, and chemogenetic approaches. To our knowledge, this is the first study to demonstrate the detailed neurobiological mechanism of placebo analgesia in chronic neuropathic pain animals. The neurobiological mechanisms of chronic pain are substantially different from those of acute pain (*79*, *80*), emphasizing that our results may provide insight for the development of effective therapeutic strategies to maximize clinical outcomes and reduce adverse drug effects in medical practice. Furthermore, placebo-related beneficial effects are apparently mediated by intrinsic neurobiological functions activated by the mind, suggesting that understanding the fundamental mechanisms of the placebo effect may provide an opportunity to study the mechanisms of self-healing capacity.

## MATERIALS AND METHODS

### Animals

All experiments were approved by Osaka University’s Animal Research Committee (approval number: 24-006-042) and the Institutional Animal Care and Use Committees of RIKEN, Kobe University, and Nihon University. Long-Evans rats for embryo donation were obtained from Japan SLC (Shizuoka, Japan). Wistar-Imamichi rats for transplant recipients of genome-edited zygotes were obtained from the Institute for Animal Reproduction, Ibaraki, Japan. All animals were housed at 23° ± 1.5°C, 45 ± 15% humidity, and a 12:12-hour light:dark cycle with a standard pellet diet (Oriental Yeast Co., Tokyo, Japan) and tap water ad libitum. Behavioral experiments were performed according to the Laboratory Animal Care Principles (National Institutes of Health publication no. 85-23, revised 1985). Behavioral studies were performed according to the Animal Research Reporting of In Vivo Experiments guidelines (*81*). All efforts were made to minimize animals used and their suffering.

### Generation of MOR-Cre KI rats by CRISPR-Cas9 genome editing

To selectively and functionally manipulate MOR^+^ neuron activity in vivo, we designed and developed genetically engineered rats expressing Cre recombinase under MOR (*Oprm1*) promoter control using CRISPR-Cas9 genome editing technology (Fig. 1A).

#### Preparation of CRISPR components and long single-stranded donor DNA

Guide RNA (gRNA) was designed using CRISPOR software (http://crispor.tefor.net/). The target sequence selected for KI production was 5′-GTGAGACCCAGTTAGGGCAA-3′. gRNA was prepared using a Precision gRNA Synthesis Kit (Thermo Fisher Scientific, Waltham, MA). Cas9 mRNA was transcribed in vitro using the mMESSAGE mMACHINE T7 Ultra Kit (Thermo Fisher Scientific) from a linearized plasmid (ID #72602; www.addgene.org/CRISPR) and purified using a MEGAClear kit (Thermo Fisher Scientific). As a KI donor, long single-stranded DNA (lssDNA) was prepared using nicking endonucleases, as reported previously (*82*). The lssDNA introduced into fertilized embryos comprised the left homology arm, T2A, nlsCre, polyA signal, and right homology arm sequences in that order (Fig. 1A).

#### Manipulation of rat embryos and electroporation

For electroporation, 100 Long-Evans embryos at 6 to 8 hours after collection were placed into a chamber with 40 μl of serum-free medium (Gibco Opti-MEM, Thermo Fisher Scientific) containing Cas9 mRNA (400 ng/μl), gRNA (200 ng/μl), and lssDNA (20 ng/μl). They were electroporated with a 5-mm gap electrode (CUY520P5; Nepa Gene, Chiba, Japan) in a NEPA21 Super Electroporator (Nepa Gene) (*83*).

Seventy-two embryos that developed to the two-cell stage after Cas9 mRNA, gRNA, and lssDNA introduction were transferred into the oviducts of three female surrogates anesthetized with isoflurane. Last, three pups were born, one of which was confirmed to be a 2A-nlsCre KI rat (Fig. 1A).

#### MOR-Cre KI genotyping and sequencing

Genotyping PCR was performed using genomic DNA extracted from the tail and KAPA2G Fast Hotstart ReadyMix with dye (KK5610; Kapa Biosystems, Wilmington, MA). PCR products amplified with specific primer sets (table S3) were directly sequenced using the BigDye terminator v3.1 cycle sequencing mix and the standard protocol for an Applied Biosystems 3130 DNA Sequencer (Applied Biosystems, Foster City, CA). The correct fragment size and sequence for the internal 5′ and 3′ junction of the transgene insertion into the target locus were validated (Fig. 1, A and B). Homozygous MOR-Cre KI rats were born at the expected Mendelian ratios and were viable and fertile without noticeable gross abnormalities.

### Viral vector production

Table S3 presents all viral vectors used in this study. AAV5-CaMKIIα-EGFP, AAV2-hSyn-DIO-mCherry, AAV2-hSyn-DIO-hM3Dq-mCherry (AAV-DIO-hM3Dq-mCherry), AAVrg-pgk-Cre (AAVrg-Cre), and AAVrg-hSyn-EGFP (AAVrg-EGFP) were purchased from Addgene. AAV5-CAG-FLEx (FRT)-TC-mCherry (AAV-fDIO-TCb-mCherry) and AAV8-CAG-FLEx (FRT)-G (AAV-fDIO-RG) were generated in the University of North Carolina at Chapell Hill vector core using plasmids (Addgene #67827 and #67828). AAV2-EF1α-Flex-hM3Dq-2A-cgfTagRFP (AAV-Flex-hM3Dq-TagRFP), AAV2-EF1α-Flex-hM4Di-2A-cgfTagRFP (AAV-Flex-hM4Di-TagRFP), AAVrg-CAGGS-IL-2Rα-EGFP (AAVrg- IL-2Rα-EGFP), AAV2-EF1α-Flex-ChR2(H134R)-mCherry (*36*) (AAV-Flex-ChR2(H134R)-mCherry), and AAVrg-EF1a-FLPo (AAVrg-FLPo) were obtained from our laboratory. These AAVs were prepared using an AAV Helper-Free System (Agilent Technologies, Santa Clara, CA) (*29*). Human embryonic kidney–293T cells were transfected with the transfer gene, replication/encapsulation gene, and adeno-helper gene plasmids using the calcium phosphate precipitation method. Two rounds of CsCl gradient ultracentrifugation were performed at 100,000*g* and 4°C for 23 hours. The fractions containing AAV were collected and subjected to three rounds of dialysis, and the dialyzed solution was concentrated by centrifugation through a Vivaspin Turbo filter (Sartorius, Gottingen, Germany). The viral genome titer of the AAV vectors was determined by quantitative PCR (TaqMan, Thermo Fisher Scientific). To prepare rabies dG-GFP + EnvA, we unitized the viruses and cell lines as described previously (*84*). RVdG-GFP was prepared de novo using B7GG cells (gift from E. Callaway) and plasmids (pCAG-B19N, pCAG-B19P, pCAG-B19L, pCAG-B19G, and pSADdG-GFP-F2; gifts from E. Callaway) and then pseudotyped using BHK-EnvA cells (gift from E. Callaway).

### Stereotaxic microinjection

Rats were anesthetized with 4 to 5% isoflurane, and anesthesia was maintained with 1.5% isoflurane. The head of each rat was fixed in a small-animal stereotaxic instrument with a Digital Display Console (Model 68025; David Kopf Instruments, Tujunga, CA). A small burr hole was drilled for microinjection. The neural tracers and AAVs (table S3) were injected into each position using a UMP3 pump regulated by Micro-4 (flow rate: 20 to 50 nl/min; World Precision Instruments, Sarasota, FL). After microinjection, the incision was sutured, an antimicrobial was injected to prevent infection, and the animal was returned to the home cage.

#### Retrograde and anterograde labeling

For retrograde somatic labeling (Fig. 4), cholera toxin subunit B (recombinant) and Alexa Fluor 647 conjugate (CTB647; Thermo Fisher Scientific) were dissolved in PBS at 1 mg/ml and microinjected into the vlPAG (200 nl). For anterograde tracing, 400 nl of AAV-CaMKIIα-EGFP was microinjected into layer V of the mPFC. The following coordinates were used: mPFC: anteroposterior (AP), +3.72; mediolateral (ML), +0.7; dorsoventral (DV), +3.2; and vlPAG: AP, −7.52; ML, +0.65; DV, +5.0.

#### Monosynaptic retrograde rabies virus tracing

To investigate whether there were direct monosynaptic connections between MOR^+^ neurons in the mPFC and layer V pyramidal neurons that project into the vlPAG (Fig. 5), 200 nl of AAVrg-FLPo was injected into the vlPAG, and 600 nl of a 1:1 mixture of AAV-fDIO-TCb-mCherry and AAV-fDIO-RG was injected into the mPFC. To label MOR^+^ neurons, 400 nl of AAV-Flex-hM4Di-TagRFP was injected into the mPFC. Two weeks after AAV injection, 200 nl of rabies dG-GFP + EnvA was injected into the mPFC. One week later, rats were perfused with 4% paraformaldehyde, and their brains were excised for further histological analysis. In the negative control (fig. S9), AAV microinjection was performed analogously, except that AAVrg-FLPo was injected into the vlPAG.

#### Selective ablation of mPFC-vlPAG circuit

We also used an ITX-mediated neural circuit elimination approach for selective ablation of the mPFC-vlPAG circuit (Fig. 7). For this purpose, 1200 nl of a mixture of AAV-Flex-hM4Di-TagRFP (900 nl) and ITX (100 nl; final, 50 ng/μl) was injected into the mPFC. AAVrg-IL-2Rα-EGFP (200 nl) was injected into the vlPAG. The following coordinates were used: mPFC: AP, +3.24/+3.00/+2.76; ML, +0.7; DV, +3.0; vlPAG: AP, −7.56; ML, +0.6; DV, +4.8.

#### Optogenetic/chemogenetic manipulation

For chemogenetic manipulation of MOR^+^ neurons in the mPFC (Figs. 2 and 3 and fig. S5), 600 nl of AAV-Flex-hM3Dq-2A-TagRFP or 1200 nl of AAV-Flex-hM4Di-TagRFP was injected into layer V of the mPFC. For chemogenetic manipulation of the mPFC-vlPAG circuit (Fig. 4 and figs. S7 and S8), 300 nl of a mixture of AAVrg-Cre (500 nl) and CTB647 (200 nl) was injected into the vlPAG, and 1200 nl of AAV-DIO-hM3Dq-mCherry or 1200 nl of AAV-Flex-hM4Di-TagRFP was injected into layer V of the mPFC.

For optogenetic activation of MOR^+^ neurons (Fig. 6, 1000 nl of AAV-Flex-ChR2(H134R)-mCherry was microinjected into the layer V of the mPFC. The following coordinates were used: mPFC: AP, +3.00; ML, ±0.7; and DV, +2.7/+3.4.

### SNI model

Neuropathic pain was induced by SNI of the unilateral sciatic nerve, as described previously (*85*). Briefly, rats (8 to 12 weeks) were anesthetized with 1.5% isoflurane, and a skin and muscle incision was made on the left hindlimb to expose the sciatic nerve and its three branches. For SNI treatment, the common peroneal and tibial nerves were ligated, and distal to the trifurcation was cut, leaving the sural nerve intact. After surgery, the incision was sutured, an antimicrobial was injected to prevent infection, and the animal was returned to the home cage. In SNI rats, the receptive field of the lateral aspect of the hindpaw skin innervated by the sural nerve displays hypersensitivity to tactile stimuli (Fig. 3, A and B).

### Measurements of pain threshold

The pain threshold in rats with neuropathic pain was measured by the up-down method using von Frey filaments, as described previously (*86*). Briefly, rats were placed in a plastic cage with a wire-mesh bottom and acclimatized for 30 to 60 min before testing. Stimulation of the bilateral hindpaws was gradually induced by von Frey filaments with 2, 4, 6, 8, 15, and 26 g of filaments (Aesthesio; DanMic Global, San Jose, CA). Stimulation was presented at intervals of at least 30 s, and the pain response was determined when rats lifted, shook, or bit the hindpaw. Pain threshold measurements started at least 5 days after SNI surgery. The PWT is calculated on the basis of a pain score, which is acquired by the pain response pattern, as 50% PWT (*86*).

### Establishment of placebo analgesia in SNI rats and chemogenetic manipulation

Placebo analgesia in rats with neuropathic pain was induced by pharmacological conditioning (*9*) using GBP hydrochloride (100 mg/kg, Tokyo Chemical International, Tokyo, Japan). As shown in Fig. 3C, the analgesic effect of GBP was significantly observed 1 hour after intraperitoneal injection and then decreased over time. The analgesic effect of a single intraperitoneal injection of GBP completely disappeared 24 hours after the injection. Pharmacological conditioning was established by pairing GBP with the intraperitoneal injection (conditioned stimulus) in SNI rats for four consecutive days. During conditioning, the analgesic effects of GBP were assessed using the von Frey filament test. Placebo analgesia was evaluated by measuring the pain threshold after an intraperitoneal saline injection as a placebo on the test day (day 5). In the chemogenetic manipulation, CNO (BML-NS105, 1 mg/kg, Enzo Life Science, Farmingdale, NY) was injected instead of saline on the test day for chemogenetic activation or suppression of specific neuronal activity.

### Histological experiments

Under deep anesthesia with isoflurane, rats were transcardially perfused with PBS, followed by 4% paraformaldehyde in PBS. Then, the brains were excised and soaked in 4% paraformaldehyde to postfix overnight at 4°C. Serial brain sections were cut at 30-μm thickness using a cryostat (Cryostar NX70, Epredia, Portsmouth, NH). For ISH, the brain sections were mounted onto matsunami hydrophilic adhesion coated slide glass (Matsunami, Osaka, Japan) and kept at −30°C.

#### Immunohistochemistry

For immunostaining, brain sections were incubated overnight at 4°C with the following primary antibodies: rabbit anti-RFP (1:1000; R10367, Thermo Fisher Scientific), mouse anti-cFos (1:500; ab208942, Abcam, Cambridge, UK), rabbit anti-mCherry (1:1000; ab167453, Abcam), rat anti-RFP monoclonal antibody (5F8) (1:1000; 5f8, Chromotek, Planegg, Germany), chicken anti-GFP (1:1000; ab13970, Abcam), and rabbit anti-GFP (1:1000; A6455, Thermo Fisher Scientific). The bound antibodies were visualized by the following secondary antibodies: Alexa Fluor–conjugated secondary antibodies (1:500; Thermo Fisher Scientific) and Jackson antibodies (1:1000; Jackson ImmunoResearch Laboratories, West Grove, PA). Brain sections were mounted on 3-aminopropyltriethoxysilane coated slide glass (Matsunami, Osaka, Japan) with ProLong Gold Antifade Mountant (Thermo Fisher Scientific) and 4′,6-diamidino-2-phenylindole (5 ng/ml; 340-07971, Dojindo, Kumamoto, Japan).

#### RNAscope ISH

ISH with RNAscope was performed according to the RNAscope Multiple Fluorescent Reagent Kit v2 Assay User manual for fresh frozen sections (#323100; ACDBio, Newark, CA). Probes targeting intronic regions for *MOR* (gene symbol: *oprm1*; ACDBio, #410691) and *Cre* (ACDBio, #312281) were synthesized. *Cre* was labeled with tyramide signal amplification (TSA) cyanine 3 (1:1000; NEL744001KT, PerkinElmer, Waltham, MA), and *MOR* was labeled with TSA fluorescein (1:1000; NEL741001KT, PerkinElmer, Waltham, MA). Fixed sections were pretreated with hydrogen peroxide for 10 min and protease III for 30 min, and the probes were hybridized and amplified according to the manufacturer’s instructions.

Double RNAscope ISH was performed in a MOR-Cre KI rat to validate the coexpression of Cre and MOR. In the mPFC regions, the coexpression ratio of Cre and MOR was counted in each cortical layer from three animals. For Fig. 1C, the counting area of each layer is defined as in previous reports (*87*, *88*).

#### Combined ISH and immunohistochemistry

For double staining of vGAT and mCherry, a combination of ISH and immunohistochemistry was performed according to our previous report (*89*). Briefly, brain sections were washed with PBS, treated with proteinase K (10 μg/ml; #9034; Takara Bio, Kusatsu, Japan), fixed with 4% paraformaldehyde, and acetylated with 0.25% acetic anhydride. After prehybridization with hybridization buffer, the vGAT RNA probe was diluted 1:1000 in hybridization buffer and applied to each slide. After 16 hours of incubation at 60°C in a humid chamber, the sections were washed with 50% formamide 2× SSC, then with 2× SSC, and lastly with 0.2× SSC twice for 20 min at 65°C. After blocking with 0.5% blocking buffer (FP1012, PerkinElmer), sections were incubated with horseradish peroxidase–conjugated anti-Dig antibody (1:500; #11207733910; Sigma-Aldrich, St. Louis, MO) overnight at 4°C. The sections were washed with phosphate-buffered saline with Tween 20 (PBST) and treated with the TSA Plus Biotin Kit (1:70; PerkinElmer, #NEL749A001KT) at room temperature for 25 min. After washing with PBST, the sections were incubated with mCherry antibody (1:1000; ab167453, Abcam) overnight at 4°C. On the fourth day, sections were washed with PBST and treated with secondary antibody Cyanine3 AffiniPure Donkey Anti-Rabbit IgG (H + L) (Jackson ImmunoResearch) for mCherry and streptavidin––Alexa Fluor 488 conjugate (Thermo Fisher Scientific, S11223). They were then washed with PBST and mounted with cover glass using ProLong Gold Antifade Mountant (Thermo Fisher Scientific).

#### Acquisition and analysis of fluorescent images

Fluorescent images were acquired using a Zeiss LSM 710 confocal microscope (Carl Zeiss, Oberkochen, Germany) and analyzed using ZEN software (Carl Zeiss). In Fig. 7 (D to G), the counting area of layer V is defined as in previous reports (*87*, *88*).

### Electrophysiology

#### Whole-cell patch-clamp

For optogenetic activation of MOR^+^ neurons in the mPFC, 500 nl of AAV-ChR2(H134R)-mCherry was microinjected into layer V of the mPFC of MOR-Cre KI rats. To identify pyramidal neurons in the mPFC that projected into the vlPAG, we injected a retrograde tracer, CTB647 (200 nl), into the vlPAG. Subsequently, 1 to 2 weeks after AAV injection, histology specimens were prepared as described previously (*90*, *91*) except for the recipe of ice-cold modified artificial cerebrospinal fluid (M-ACSF) (*92*). Briefly, rats were deeply anesthetized with isoflurane (5%) and perfused transcardially with ice-cold M-ACSF [2.5 mM KCl, 0.5 mM CaCl_2_, 10 mM MgSO_4_, 1.25 mM NaH_2_PO_4_, 2 mM thiourea, 3 mM sodium pyruvate, 92 mM *N*-methyl-d-glucamine, 20 mM Hepes, 25 mM d-glucose, 5 mM l-ascorbic acid, and 30 mM NaHCO_3_ (pH 7.35 to 7.40)]. After decapitation, the tissue blocks, including the mPFC, were rapidly removed and stored in ice-cold M-ACSF. Coronal slices were cut at a thickness of 350 μm using a microslicer (Linearslicer Pro 7; Dosaka EM, Kyoto, Japan) and were incubated in holding ACSF at 32°C [2.5 mM KCl, 2 mM CaCl_2_, 2 mM MgSO_4_, 1.25 mM NaH_2_PO_4_, 2 mM thiourea, 3 mM sodium pyruvate, 92 mM NaCl, 20 mM Hepes, 25 mM d-glucose, 5 mM l-ascorbic acid, and 30 mM NaHCO_3_ (pH 7.35 to 7.40)]. The slices were then transferred to a recording chamber containing normal ACSF (N-ACSF) at room temperature (126 mM NaCl, 3 mM KCl, 2 MgSO_4_, 1.25 mM NaH_2_PO_4_, 26 mM NaHCO_3_, 2 mM CaCl_2_, and 10 mM d-glucose). All ACSF solutions were continuously aerated with a mixture of 95% O_2_ and 5% CO_2_.

Thin-wall borosilicate patch electrodes (3 to 5 megohm) were pulled using a Flaming-Brown micropipette puller (P-97, Sutter Instruments, Novato, CA). The pipette solution contained the following components (*E*_Cl_^−^ = −65 mV): 135 mM potassium gluconate, 10 mM Hepes, 0.5 mM EGTA, 2 mM MgCl_2_, 2 mM Mg–adenosine triphosphate, and 0.3 mM Na–guanosine triphosphate. The pipette solution had a pH of 7.3 and an osmolarity of 300 mOsm. The liquid junction potential of the pipette solution described above was −9 mV. Voltage was not corrected in this study.

For recording, the slices, including the mPFC, were placed in a recording chamber perfused continuously with N-ACSF at 2.4 ml/min. The recordings were obtained at room temperature. The seal resistance was >10 gigohm, and only data obtained from electrodes with access resistance of 6 to 20 megohm and with <20% change during recordings were included. Alexa Fluor 488 (Thermo Fisher Scientific) was added to the internal solution in a subset of experiments to identify the neural subtypes of the recorded neurons (Fig. 6).

Whole-cell patch-clamp recordings were obtained from mCherry^+^ or CTB^+^ neurons located in layer V using a fluorescence microscope equipped with Nomarski optics (BX61W1; Olympus) and an infrared-sensitive video camera (IR-1000; Dage-MTI, Michigan City, IN). Electrical signals were recorded with an amplifier (Multiclamp 700B, Molecular Devices, San Jose, CA) and a digitizer (Axon Digidata 1440A, Molecular Devices), observed online, and stored on a computer hard disk using Clampex (pClamp 10, Molecular Devices).

The voltage responses of mCherry^+^ or CTB^+^ neurons were recorded by injecting depolarizing and hyperpolarizing current pulses (500 ms) to examine basic membrane properties, including input resistance, single-spike kinetics, voltage-current relationship, and repetitive firing patterns and frequency.

To examine photostimulation-evoked synaptic responses, ChR2, expressed in MOR^+^ neurons, was activated by collimated blue light (470 nm) using a light-emitting diode system (8.6 mW) through a water-immersion 40× microscope objective. Photostimulation was applied to the slices for 1 to 5 ms. We first recorded synaptic responses to single and 15-train photostimulation under the current-clamp condition. Next, photostimulation-evoked synaptic responses were recorded under voltage-clamp conditions, in which the holding potentials were set at −60 or −40 mV.

To determine whether the photostimulation-evoked synaptic responses were monosynaptic or polysynaptic, we applied tetrodotoxin (1 μM, Abcam), 4-AP (1 mM; Nacalai Tesque, Kyoto, Japan), and picrotoxin (100 μM; Sigma-Aldrich). To examine the effects of activating MORs, we administered DAMGO, a MOR agonist, with or without CTAP, a selective MOR antagonist. All drugs were bath applied to the perfusate. The other compounds were purchased from Wako Pure Chemical Industries. All recorded data were analyzed using Clampfit (pClamp 10, Molecular Devices). The average amplitude was obtained from 10 to 20 consecutive sweeps.

#### In vivo extracellular multichannel recordings

To confirm the chemogenetic activation of MOR^+^ neurons, we also recorded the extracellular action potential in the mPFC of MOR-Cre KI rats in which AAV-Flex-hM3Dq-TagRFP was microinjected into layer V of the mPFC (fig. S4). At least 2 weeks after the AAV injection, the rats were anesthetized with 1.8% isoflurane, and the heads were fixed in a stereotaxic instrument (Model 68025; David Kopf Instruments). A 32-channel silicon probe (A4 × 8–5 mm–100–200–177, with eight electrodes in a linear arrangement on four shanks; NeuroNexus Technologies, Ann Arbor, MI) was inserted vertically into the mPFC using a micromanipulator on a stereotaxic frame. The silicon probe was left in place for 30 min to allow the brain tissue to recover from the acute damage. Then, a 10-min extracellular multichannel action potential recording was performed before and after CNO injection (1.0 mg/kg, ip). The 10 min of recordings was repeated every 30 min until 160 min after CNO injection. Extracellular signals were amplified and filtered using a 32-channel head stage (band pass: 0.3 to 7.5 kHz; Cereplex M, Blackrock Microsystems, Salt Lake City, UT). Amplified signals were digitized at 30 kHz using a head stage. Extracellular signals were processed offline to isolate spike events of individual neurons using the spike-sorting software BOSS (Blackrock Microsystems).

### Statistical analysis

All data were analyzed using GraphPad Prism version 8 (GraphPad Software, San Diego, CA) and are presented as mean ± SEM. We used the two-way repeated-measures analysis of variance (ANOVA) followed by Bonferroni’s comparison, one-way ANOVA followed by Tukey’s multiple comparisons, the unpaired *t* test, and the unpaired *t* test with Welch’s correction. Differences with *P* values of <0.05 were considered statistically significant. All statistical data are presented in table S2.
