# Supplementary material for: Opioidergic activation of the descending pain inhibitory system underlies placebo analgesia
Source: Sci Adv. 2025 Jan 15;11(3):eadp8494. doi: 10.1126/sciadv.adp8494 (PMC11734720; doi:10.1126/sciadv.adp8494)
Supplement: Supplementary file 1 — Figs. S1 to S9 Tables S1 to S3 [file sciadv.adp8494_sm.pdf]

Science Advances
AAAS

Supplementary Materials for

Opioidergic activation of the descending pain inhibitory system underlies placebo analgesia

Hiroyuki Neyama *et al.*

Corresponding author: Yilong Cui, cuiyl@riken.jp

Sci. Adv. **11**, eadp8494 (2025)
DOI: 10.1126/sciadv.adp8494

This PDF file includes:

Figs. S1 to S9
Tables S1 to S3

Fig. S1.

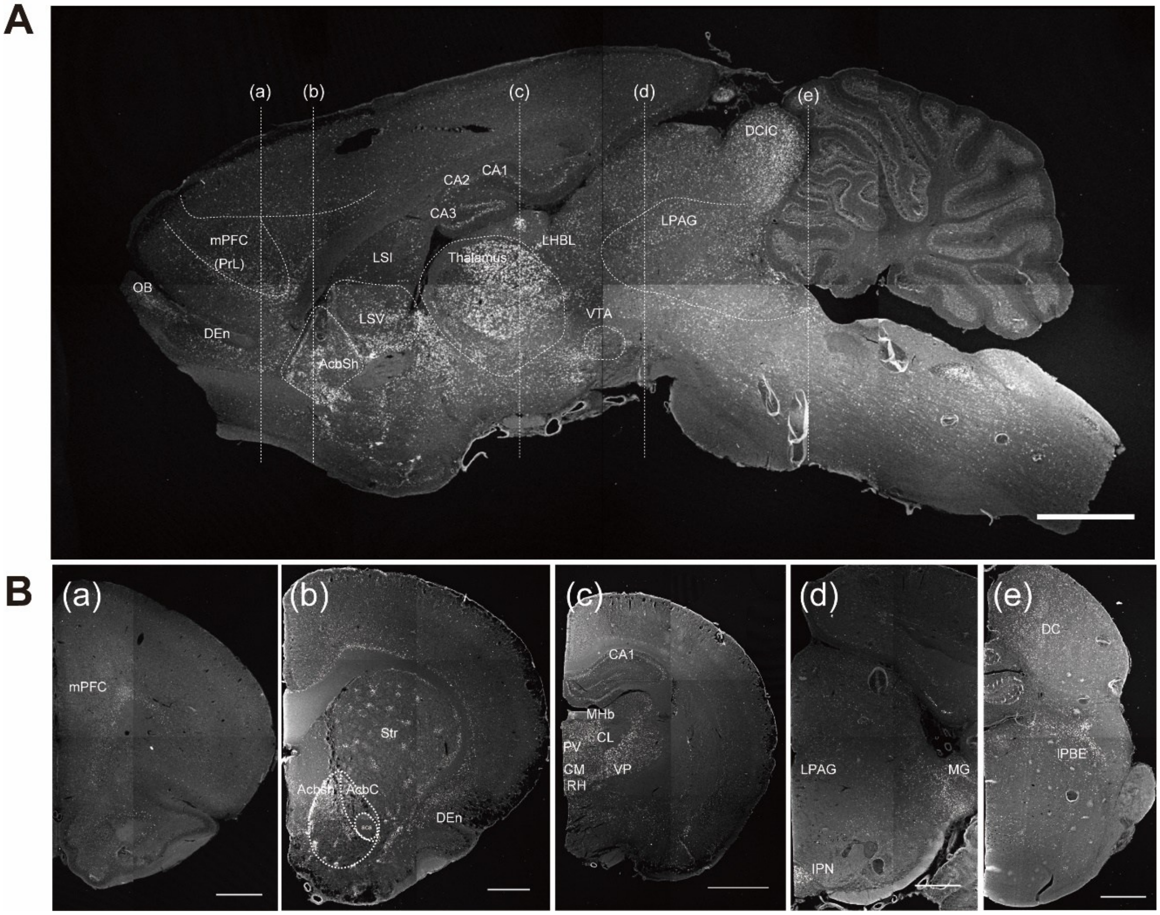

Fig. S1. Distribution of MOR gene expression by RNAscope. (**A**) Distribution of MOR gene expression in sagittal brain section. Acbsh; nucleus accumbens Shell, CA1/2/3; hippocampus, dHP; dorsal hippocampus, DCIC; dorsal cortex of the inferior colliculus, DEn; dorsal endopiriform nucleus, LHBL; lateral habenula nucleus, lateral part, LSI; lateral septal nucleus, intermediate part, LSV; lateral septal nucleus, ventral part, LPAG; lateral periaqueductal gray, mPFC; medial prefrontal cortex; PrL; prelimbic cortex, OB; olfactory bulb, VTA; ventral tegmental area. (**Ba**) MOR gene expression in coronal brain section in cut surface (**a**) of A, including a mPFC of PrL (**Bb**) MOR gene expression in coronal brain section in cut surface (**b**) of A, including striatum (Str), Acbsh, nucleus accumbens core (AcbC), and DEn. (**Bc**) MOR gene expression in coronal brain section in cut surface (**c**) of A, including the thalamus (central medial thalamic nucleus (CM), centrolateral thalamic nucleus (CL), ventral posterior thalamic nucleus (VP), praventricular thalamic nucleus (PV), rhomboid thalamic nucleus (RH)), medial habenula (MHb), and hippocampus (CA1). (**Bd**) MOR gene expression in coronal brain section in cut surface (**d**) of A, including LPAG, Interpeduncular nucleus (IPN), and medial geniculate nucleus (MG). (**Be**) MOR gene expression in coronal brain section in cut surface (**e**) of A, concluding DC and lateral parabrachial nucleus, eternal part (lPBE). Scale bars: (**A**) 2000 μm, (**Ba**) 1000 μm, (**Bb**) 1000 μm, (**Bc**) 2000 μm, (**Bd**) 1000 μm, (**Be**) 1000 μm.

Fig. S2

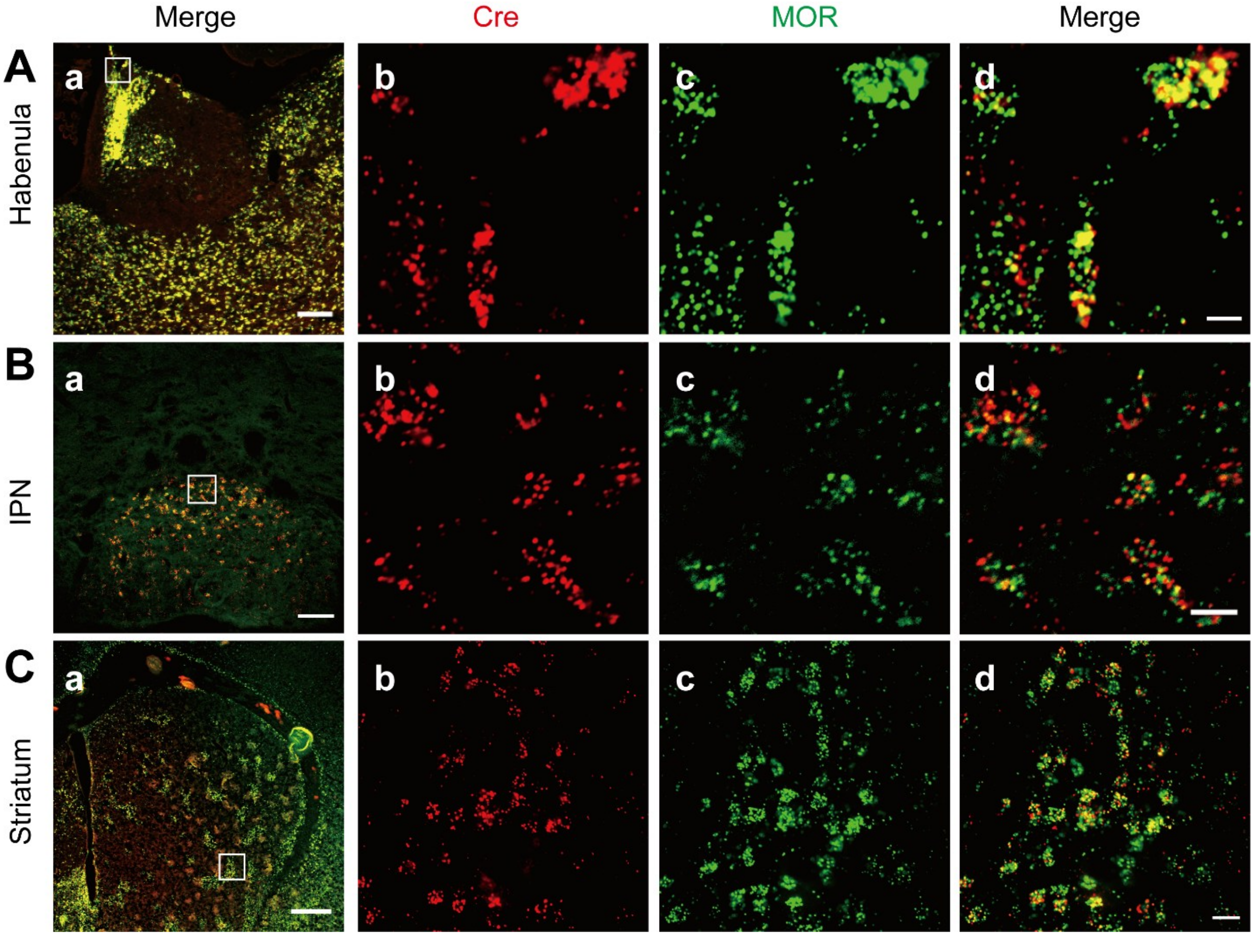

Fig. S2. Co-expression of Cre and MOR in MOR-enriched brain regions. (**A** to **C**) Double RNAscope fluorescent images of Cre and MOR in the habenula (**A**), interpeduncular nucleus (IPN; **B**), and striatum (**C**). (**a**) Low magnification image for co-expression with Cre and MOR. Scale bar: (**Aa**) 200 μm, (**Ba**) 100 μm, (**Ca**) 500 μm. (**b to d**) High magnification images for (**b**) Cre, (**c**) MOR, and (**d**) co-expression, respectively, Scale bar: (**Ad** and **Bd**) 10 μm, (**Cd**) 20 μm.

Fig. S3

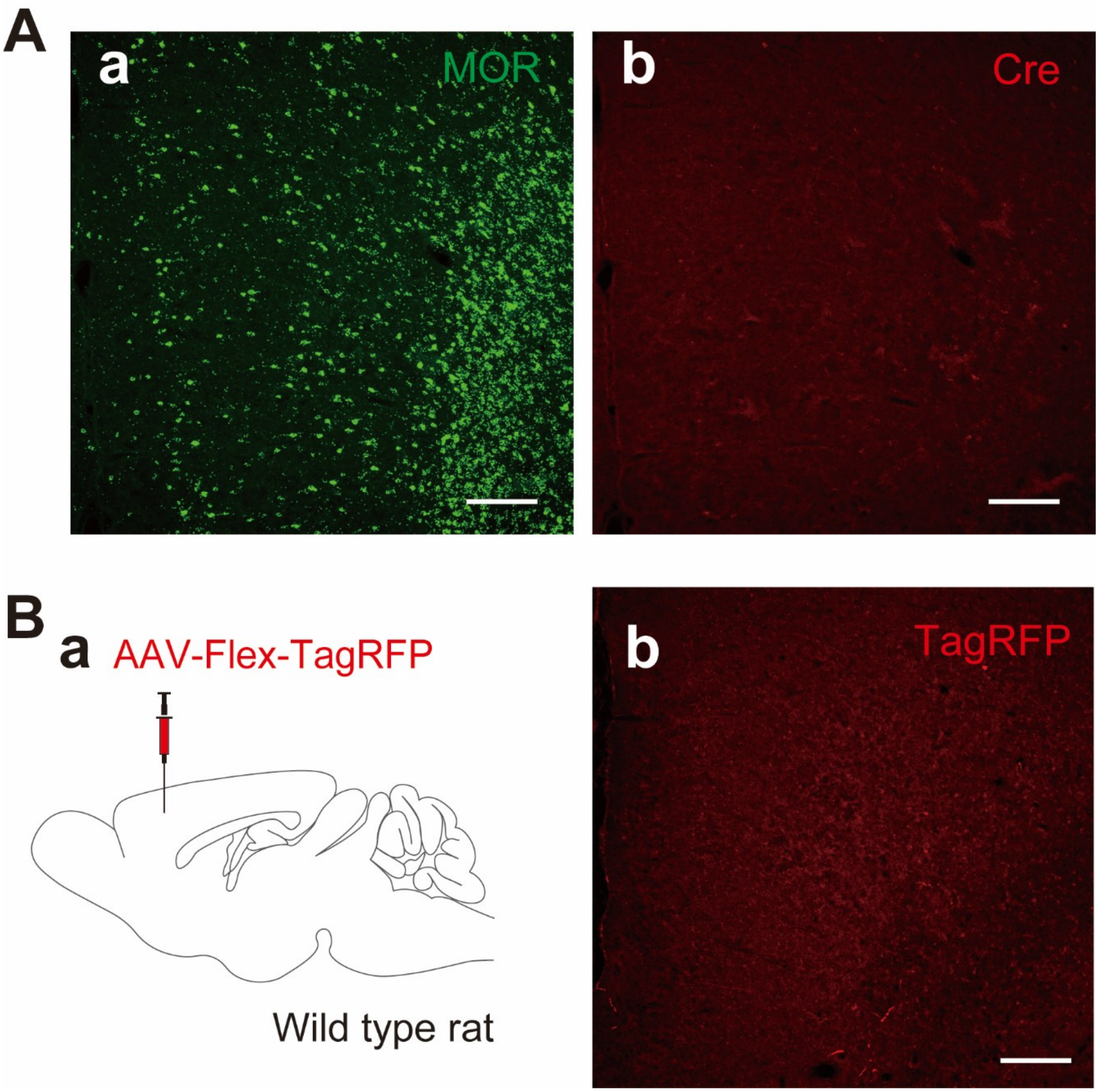

Fig. S3. MOR-specific expression of Cre gene in MOR-Cre knock-in rat. (**A**) No expression of Cre gene by RNAscope in wild-type rats. (**B**) Immunostaining image of TagRFP in wild-type rats injected with AAV-Flex-hM4Di-TagRFP into medial prefrontal cortex. Scale bar: 200 μm

Fig. S4

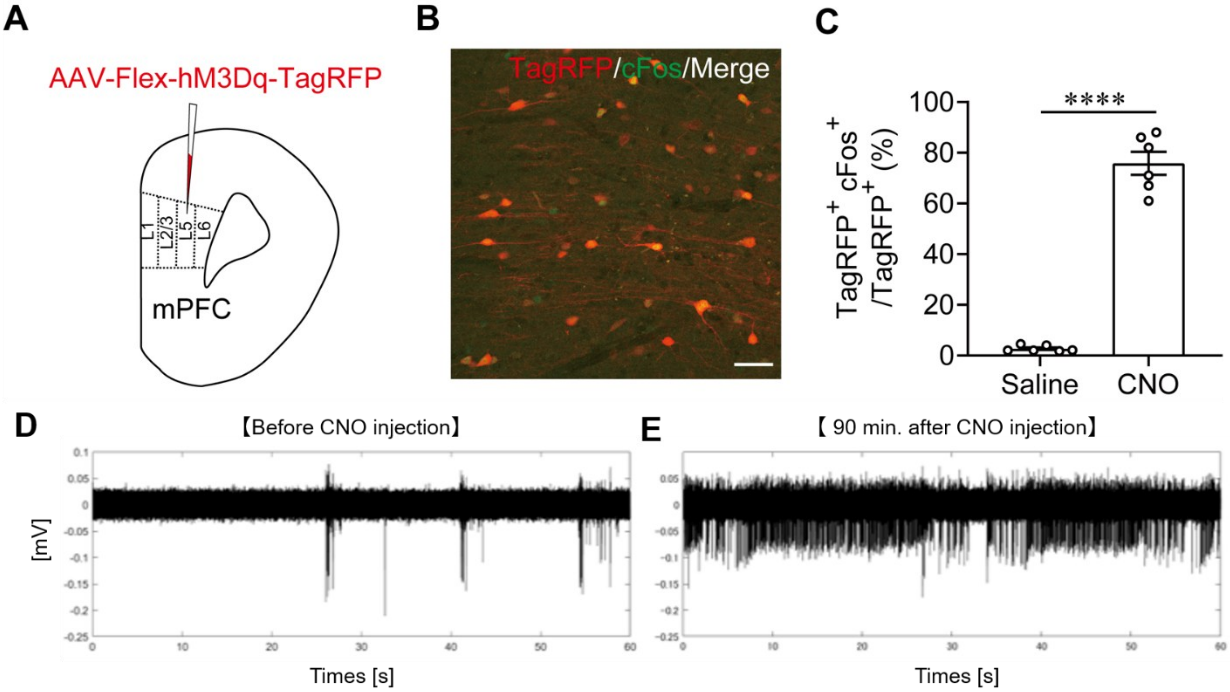

Fig. S4. Evidence of neural activation by CNO injection in a Gq-DREADD experiment. (**A**) Experimental procedure for AAV-Flex-hM3Dq-TagRFP injection. (**B**) Immunostaining image of TagRFP (red) and cFos (green) at 90 min after clozapine-*N* oxide (CNO; 1 mg/kg, intraperitoneal) injection in MOR-Cre KI rat injected with AAV-Flex hM3Dq-TagRFP into the mPFC. Scale bar: 50 µm. (**C**) Percentage of $TagRFP^+$ (MOR^+ neuron) and cFos co-expression. (**D**) Representative single-unit activities of MOR-Cre KI rat in mPFC before CNO injection. (**E**) Representative single-unit activities of MOR-Cre KI rat in mPFC 90 min after CNO (1 mg/kg, intraperitoneal) injection. Note that approximately 9.7 % of single-unit activity was increased and 10.3% was decreased variably in response to the CNO injection. ****P<0.0001. All statistical information is presented in Table S2.

Fig. S5

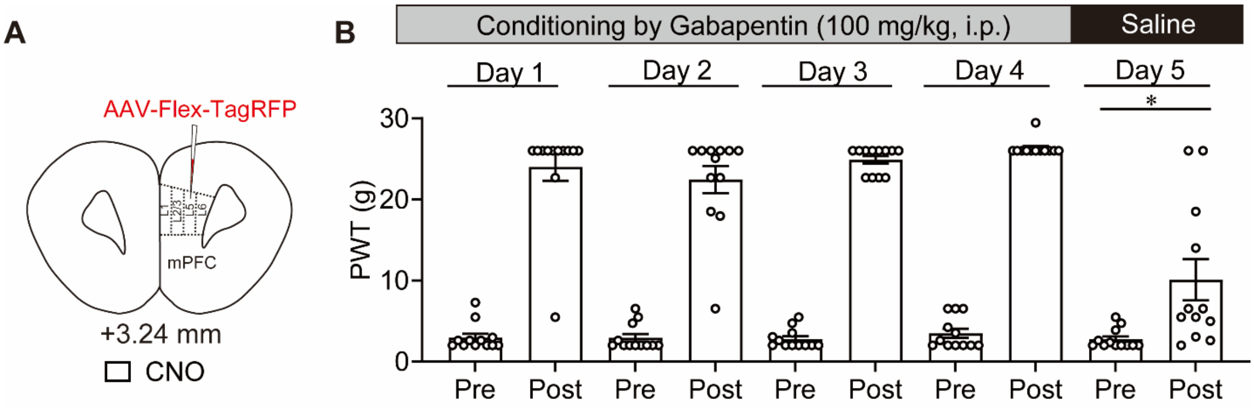

Fig. S5. Control experiment for Gq-DREADD in conditioning-induced placebo analgesia. (**A**) Experimental procedure of AAV-Flex-TagRFP (control vector) into medial prefrontal cortex for placebo experiments. (**B**) Change in paw withdrawal threshold at 60 min after gabapentin hydrochloride (conditioning, Day 1–Day 4) and saline (placebo test, Day 5). *$P<0.05$. All statistical information is presented in Table S2.

Fig. S6

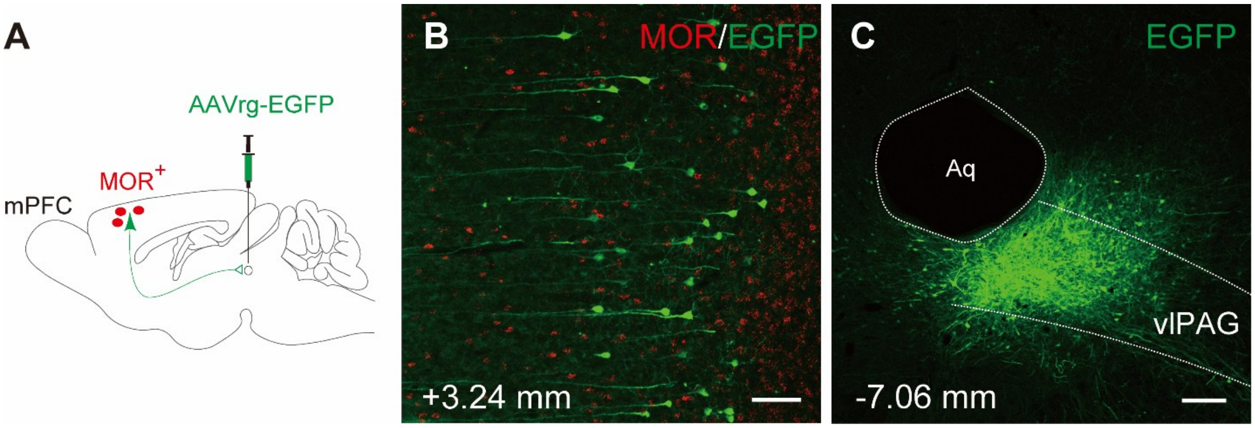

Fig. S6. Absence of co-expression of MOR and EGFP in layer-V pyramidal neuron to vlPAG. (**A**) Experimental procedure for investigating a colocalization with MOR$^+$ neuron (red color) and pyramidal neuron projection to ventrolateral periaqueductal gray (vlPAG, showing green color). (**B**) Fluorescent image using RNAscope and immunohistochemistry of GFP. (**C**) Injection area of AAVrg-EGFP in vlPAG. Scale bar: (**B**) 100 µm, (**C**) 200 µm.

Fig. S7.

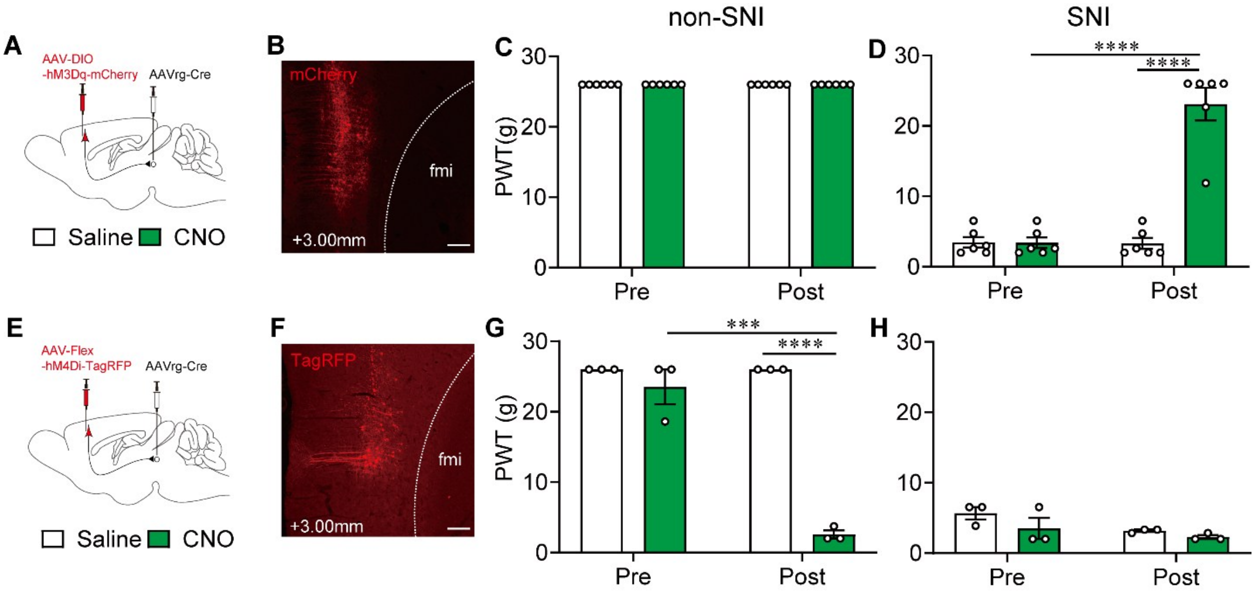

Fig. S7. Involvement of the mPFC-vlPAG pathway in pain modulation. (**A**) Experimental procedure for Gq-DREADD in non-spared nerve injury (SNI) and SNI rats. (**B**) Immunostaining image for the expression of mCherry in medial prefrontal cortex (mPFC). (**C** and **D**) Change in paw withdrawal threshold (PWT) after saline (white column) and clozapine-*N*-oxide (CNO, 1 mg/kg, intraperitoneal; green column) in non-SNI (**C**) and SNI (**D**) Rats injected with AAV-DIO-hM3Dq-mCherry. (**E**) Experimental procedure for Gi-DREADD in non-SNI and SNI rats. (**F**) Immunostaining image for the expression of TagRFP in mPFC. (**G** and **H**) Change in PWT after saline (white column) and CNO, 1 mg/kg, intraperitoneal (green column) in non-SNI (**G**) and SNI (**H**) rats with AAV-Flex-hM4Di-TagRFP. Scale bar: 200 μm. ****P*<0.001, *****P*<0.0001. All statistical information is presented in Table S2.

Fig. S8.

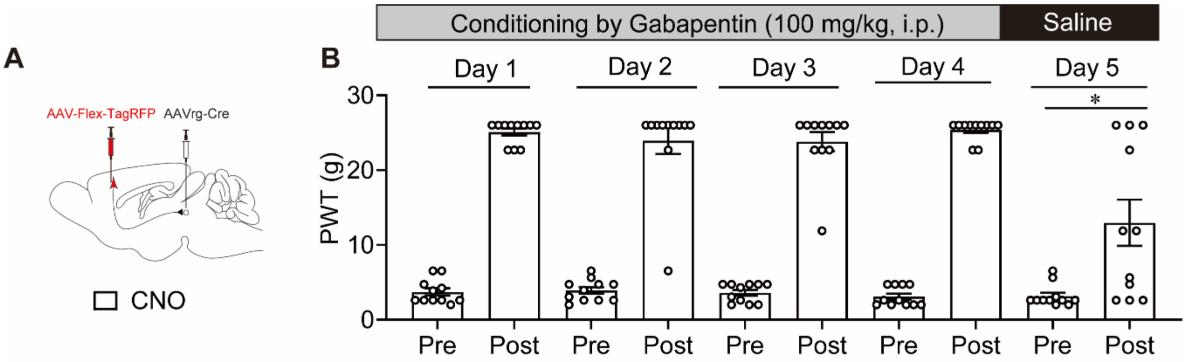

Fig. S8. Control experiment for Gi-DREADD in conditioning-induced placebo analgesia. (**A**) Experimental procedure of AAV-Flex-TagRFP (control AAV vector) in the medial prefrontal cortex for placebo experiments. (**B**) Change in paw withdrawal threshold at 60 min after gabapentin hydrochloride (conditioning, Day 1–Day 4) and saline (placebo test, Day 5). **P*<0.05. All statistical information is presented in Table S2.

Fig. S9.

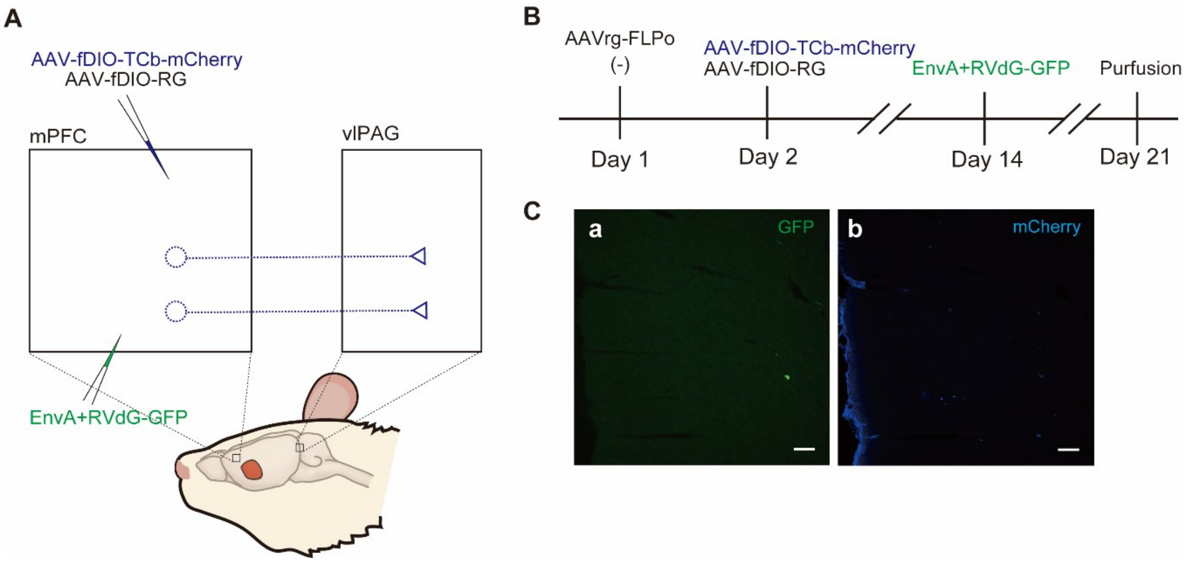

Fig. S9. Control experiment for monosynaptic retrograde rabies virus tracing in Figure 5. (**A**) Experimental procedures in the control experiment of monosynaptic retrograde rabies virus tracing. (**B**) Experimental schedule for the control experiments of monosynaptic retrograde rabies virus tracing. (**C**) Immunostaining for (**a**) GFP and (**b**) mCherry. Scale bar: 100 μm.

Table S1. Number of embryos transferred and KI pups obtained

1-cell embryo electroporated	2-cell embryo transferred	Pups delivered	KI pups
100	72	3	1

KI, knock-in

Table S2. Statistical information for all figures

Figure Number		Primary	Post-hoc	Number of animals
Fig. 2J	SNI (-) Stimuli (+) *vs.* SNI (+) stimuli (+)<br>SNI (+) Stimuli (-) *vs.* SNI (+) stimuli (+)	One-way ANOVA	Tukey	SNI (-) Stimuli (+) n=3<br>SNI (+) Stimuli (-) n=5<br>SNI (+) stimuli (+) n=6
Fig. 2L	<u>Post group</u><br>Saline *vs.* CNO<br><u>CNO group:</u><br>Pre *vs.* Post	Two-way RM ANOVA	Bonferroni	Saline n=8<br>CNO n=7
Fig. 2O	<u>Post group</u><br>Saline *vs.* CNO<br><u>CNO group</u><br>Pre *vs.* Post	Two-way RM ANOVA	Bonferroni	Saline n=6<br>CNO n=6
Fig. 3B	Day 3: Sham *vs.* SNI<br>Day 7: Sham *vs.* SNI<br>Day 10: Sham *vs.* SNI<br>Day 14: Sham *vs.* SNI	Two-way RM ANOVA	Bonferroni	Sham n=5<br>SNI n=5
Fig. 3C	<u>1 hour after injection</u><br>Saline<br>*vs.*<br>GBP 100 mg/kg	Two-way RM ANOVA	Bonferroni	Saline n=3<br>GBP n=3
Fig. 3E	Pre vs. Post	One-way RM ANOVA	Bonferroni	n=10
Fig. 3F	Pre vs. Post	One-way RM ANOVA	Bonferroni	n=10
Fig. 4M	<u>Post group</u><br>Saline *vs.* CNO<br><u>Saline group</u><br>Pre *vs.* Post	Two-way RM ANOVA	Bonferroni	Saline n=12<br>CNO n=12
Fig. 7H	PBS *vs.* ITX	Unpaired t-test		PBS n=5<br>ITX n=6
Fig. 7K	<u>Post group</u><br>PBS/hM4Di<br>*vs.*	Two-way RM ANOVA	Bonferroni	PBS/hM4Di n=5<br>ITX/hM4Di n=6

	ITX/hM4Di<br><u>PBS/hM4Di group</u><br>Pre *vs.* Post			
Fig. S4C	Saline *vs.* CNO	Unpaired t-test with Welch's correction		Saline n=6<br>CNO n=6
Fig. S5B	Day 5<br>Pre *vs.* Post	Unpaired t-test with Welch's correction		n=12
Fig. S7D	<u>Post group</u><br>Saline *vs.* CNO<br><u>CNO group</u><br>Pre *vs.* Post	Two-way RM ANOVA	Bonferroni	Saline n=6<br>CNO n=6
Fig. S7G	<u>Post group</u><br>Saline *vs.* CNO<br><u>CNO group</u><br>Pre *vs.* Post	Two-way RM ANOVA	Bonferroni	Saline n=3<br>CNO n=3
Fig. S8B	<u>Day 5</u><br>Pre *vs.* Post	Unpaired t-test with Welch's correction		n=11

ANOVA, analysis of variance; RM, repeated measures; vGAT, vesicular GABA transporter; CNO, clozapine-*N*-oxide; PBS, phosphate-buffered saline; ITX, immunotoxin; GBP, gabapentin hydrochloride; SNI, spared nerve injury.

Table 3. Reagent resources

Reagent	Source	Identifier
**Viral vectors and plasmid**		
AAV2-EF1α-Flex-hM3Dq-2A-cgfTagRFP	Fukushima Medical Univ.<br>Dr. Kobayashi Lab.	N/A
AAV2-EF1α-Flex-hM4Di-2A-cgfTagRFP	Fukushima Medical Univ.<br>Dr. Kobayashi Lab.	N/A
AAV2-EF1α-Flex-2A-cgfTagRFP	Fukushima Medical Univ.<br>Dr. Kobayashi Lab.	N/A
AAVrg-CAGGS-IL-2α-EGFP	Fukushima Medical Univ.<br>Dr. Kobayashi Lab.	N/A
AAV2-EF1α-Flex-ChR2(H134R)-mCherry (36)	Fukushima Medical Univ.<br>Dr. Kobayashi Lab.	N/A
AAVrg-EF1α-FLPo	Riken BDR<br>Dr. Miyamichi Lab.	N/A
AAV5-CAG-FLEx (FRT)-TC-mCherry	Addgene<br>(Watertown, MA)	67827
AAV8-CAG-FLEx (FRT)-G	Addgene	67828
Rabies dG-GFP+EnvA	Riken BDR<br>Dr. Miyamichi Lab	None
AAV5-CaMKIIα-EGFP	Addgene	50469
AAV2-hSyn-DIO-hM3Dq-mCherry	Addgene	44361
AAV2-hSyn-DIO-mCherry	Addgene	50459
Retrograde AAV-pgk-Cre (AAVrg-Cre)	Addgene	24593
Retrograde AAV-hSyn-EGFP (AAVrg-EGFP)	Addgene	50465
linearized plasmid	Addgene	72602
**Antibodies**		

Rabbit anti-RFP	Thermo Fisher Scientific (Waltham, MA)	R10367 RRID: AB_10563941
Mouse anti-c-Fos	Abcam (Cambridge, UK)	ab208942 RRID: AB_2747772
Rabbit anti-mCherry	Abcam	ab167453 RRID: AB_2571870
Rat anti-RFP antibody [5F8]	Chromotek (Planegg, Germany)	5f8 RRID: AB_2336064
Chicken anti-GFP	Abcam	ab13970 RRID: AB_300798
Rabbit anti-GFP	Thermo Fisher Scientific	A6455 RRID: AB_2314549
Donkey anti-Mouse IgG (H+L) Highly Cross-Adsorbed Secondary Antibody, Alexa Fluor™ Plus 488	Thermo Fisher Scientific	A32766 RRID: AB_2762823
Donkey anti-Rabbit IgG (H+L) Highly Cross-Adsorbed Secondary Antibody, Alexa Fluor™ Plus 488	Thermo Fisher Scientific	A32790 RRID: AB_2762833
Donkey anti-Rabbit IgG (H+L) Highly Cross-Adsorbed Secondary Antibody, Alexa Fluor™ Plus 568	Thermo Fisher Scientific	A10042 RRID: AB_2534017
Cy™5 AffiniPure™ Donkey Anti-Rat IgG (H+L)	Jackson ImmunoResearch (West Grove, PA)	712-175-153 RRID: AB_2340672
Cy™3 AffiniPure Donkey Anti-rabbit IgG (H+L)	Jackson ImmunoResearch	715-165-152
Cy™2 AffiniPure Donkey Anti-chicken IgY (IgG) (H+L)	Jackson ImmunoResearch	703-225-155
**RNAscope**		
RNAscope® Multiple Fluorescent Reagent Kit v2 Assay User manual for Fresh Frozen sections	Advanced Cell Diagnostics (Newark, CA)	#323100
RNAscope Probe-Mu opioid receptor 1 (*oprm1*), MOR	Advanced Cell Diagnostics	#410691
RNAscope Probe-Cre	Advanced Cell Diagnostics	#312281
TSA Cyanine 3	Perkin Elmer (Waltham, MA)	NEL744001KT

TSA Fluorescein	Perkin Elmer	NEL741001KT
**In situ hybridization**		
Proteinase K	Takara Bio inc.<br>(Kusatsu, Japan)	#9034
Blocking regent	Perkin Elmer	FP1012
Horseradish peroxidase (HRP)-conjugated anti-Dig antibody	Sigma Aldrich	#11207733910
TSA Plus Biotin Kit	Perkin Elmer	#NEL749A001 KT
mCherry antibody	Abcam	ab167453
Cy™3 AffiniPure™ Donkey Anti-Rabbit IgG (H+L)	Jackson ImmunoResearch	715-165-152
Streptavidin-Alexa Fluor™ 488 Conjugate	Thermo Fisher Scientific	S11223
**Knock-in animal production**		
Precision gRNA Synthesis Kit	Thermo Fisher	A29377
mMESSAGE mMACHINE T7 Ultra Kit	Thermo Fisher	AM1345
KAPA2G Fast Hotstart ReadyMix with dye	Kapa Biosystems<br>(Wilmington, MA)	KK5610
**Genotyping Primers**		
F1: 5’-TGATGGCCCTCATTCTGAGC-3’ (R1)	Thermo Fisher Scientific	N/A
F2: 5’-GGCGCTAAGGATGACTCTGG-3’ (R2, 3)	Thermo Fisher Scientific	N/A
F3: 5’-TAGTTGCCAGCCATCTGTTG-3’ (F1)	Thermo Fisher Scientific	N/A
R1: 5’-TAACTACCTGTTTTGCCG GG-3’ (F2)	Thermo Fisher Scientific	N/A
R2: 5’-GACAGCAAGGGGGAGGAT-3’ (F2, 3)	Thermo Fisher Scientific	N/A
R3: 5’-ATGTGGGGGTTAGGAGAGGG -3’	Thermo Fisher Scientific	N/A
**DREADD experiment**		
Clozapine-N-oxide	Enzo Life Science<br>(Farmingdale, NY)	BML-NS105
**Neural Tracing**		

Cholera toxin subunit B (Recombinant), Alexa Fluor^{TM} 647	Thermo Fisher Scientific	C34778
**Whole-cell Patch-clamp**		
tetrodotoxin	Abcam	Ab120055
4-aminopyridine	Nacalai Tesque (Kyoto, Japan)	504-24-5
picrotoxin	Sigma-Aldrich	124-87-8
DAMGO	Wako (Richmond, VA)	78123-71-4
CTAP	Wako	103429-32-9
